# Fatty aldehyde bisulfite adducts as a purification handle in ionizable lipid synthesis†

**DOI:** 10.1039/d4ra05189k

**Published:** 2024-08-19

**Authors:** Graham Atwood, Sona Purbiya, Cassandra Reid, Brandon Smith, Kuljit Kaur, Drew Wicks, Peter Gaudet, K. Cory MacLeod, Jean-Francois Vincent-Rocan

**Affiliations:** a BioVectra Inc. Charlottetown Prince Edward Island C1E 0A1 Canada jvincent-rocan@biovectra.com

## Abstract

Rapid access to ALC-0315, a crucial component of the formulated Pfizer Covid vaccine, was obtained by employing solid adduct formation and filtration after an oxidation step in place of the standard chromatographic separation, allowing for a more scalable synthesis. Impurities were removed by formation of this fatty aldehyde bisulfite adduct at the penultimate step and by performing the final reductive amination directly with the fatty aldehyde bisulfite adduct. This eliminates chromatographic separations for all prepared aldehyde containing intermediates. Along with ALC-0315, FTT5 and SM-102 ionizable lipids were prepared utilizing this strategy. This work paves the way for more sustainable access to these critical ionizable lipids that would de-risk the world supply of important vaccines and medicines in the future.

## Abstract 2

Rapid access to ALC-0315, a crucial component of the formulated Pfizer Covid vaccine, was obtained by employing solid adduct formation and filtration in place of chromatographic separation, allowing for a more scalable synthesis.

## Graphical abstract

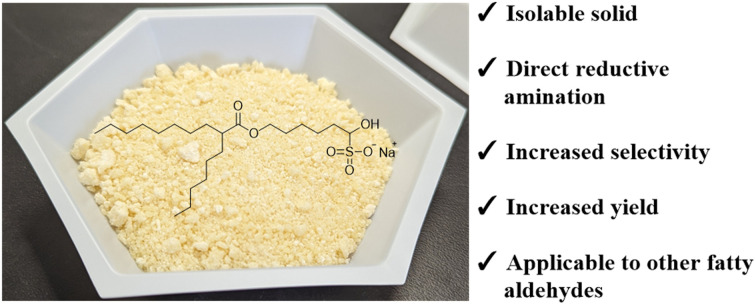

## Introduction

Lipid nanoparticles (LNPs) play a crucial role in the formulation of nucleic acid derived medicines. Ionizable lipids are the major component of these LNPs, and their chemical and physical properties are responsible for the entrapment and delivery of the payload.^1–5^ These lipids are required in significantly larger quantities than the nucleic acids they carry. However, their synthesis includes major drawbacks from a process chemistry perspective such as excessive use of chlorinated solvents and multiple column chromatography operations. ALC-0315 1 is the ionizable lipid in the Pfizer-BioNTech Covid-19 vaccine, Comirnaty, (0.43 mg per dose) and an estimated 4.6 billion doses were shipped to 181 countries.^6^ Therefore, since 2020 more than 2 MT of ALC-0315 was produced. Herein, we present our effort to make ionizable lipid production more sustainable by developing a route that relies on bisulfite adduct formation and filtration rather than chromatographic purification of intermediates to obtain analytically pure lipid.

**Scheme 1:**
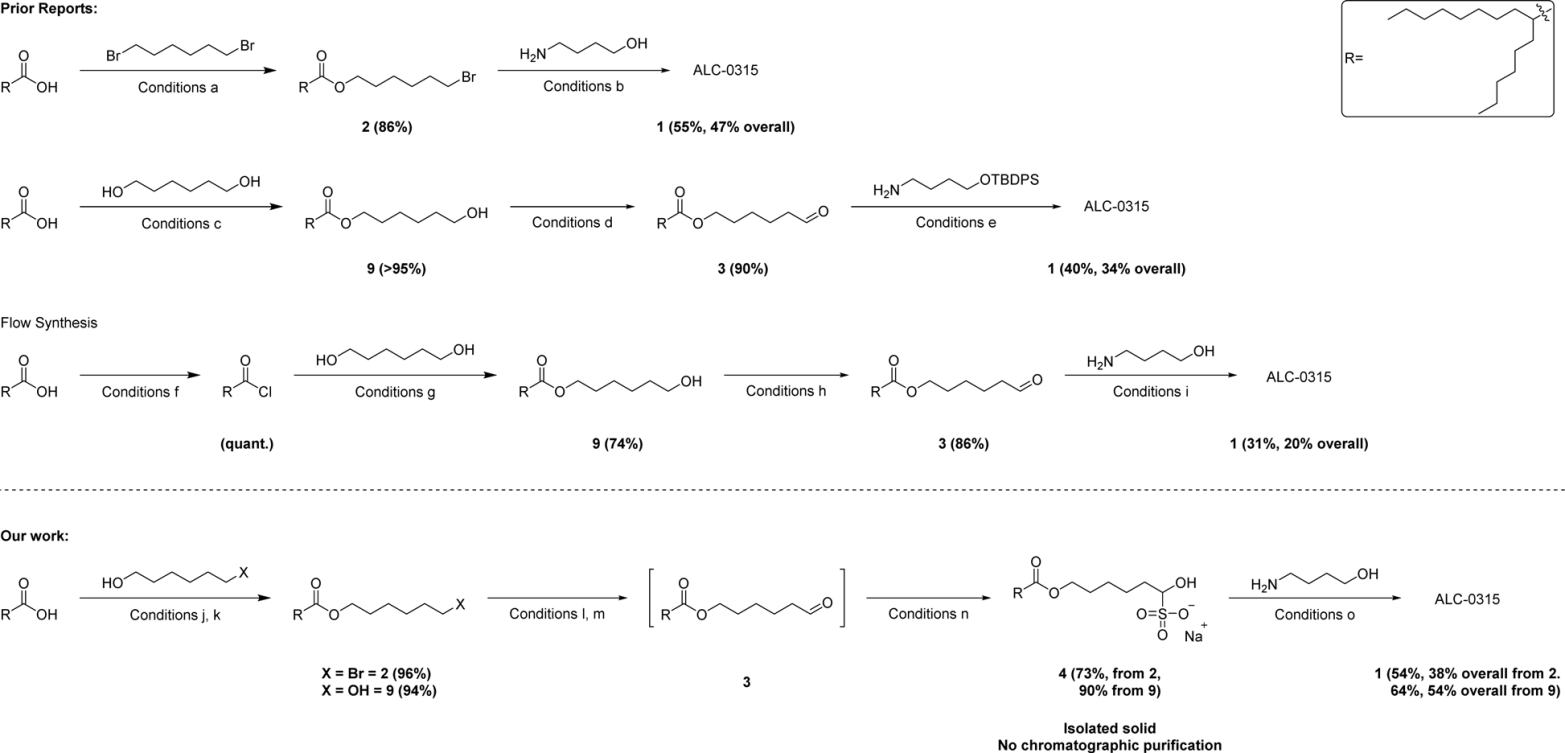
Comparison of prior reports and our work in the synthesis of ALC-0315 1. Overall yields are with respect to hexyldecanoic acid (acid). (a) Acid (1 equiv.), 1,6-dibromohexane (4 equiv.), K_2_CO_3_ (1.5 equiv.), DMF, 72 h.^7^ (b) 2 (2.2 equiv.), 4-aminobutanol (1 equiv.), K_2_CO_3_ (2.2 equiv.), 72 h, DMF.^7^ (c) Acid (1 equiv.), DCC (1.1 equiv.), DMAP (1.2 equiv.), CH_2_Cl_2_, 72 h.^8^ (d) 9 (1 equiv.), TEMPO (0.2 equiv.), bleach, NaHCO_3_ aq. (sat.), CH_2_Cl_2_, 2 h. (e) 3 (2.3 equiv.), Na(CH_3_CH_2_CO_2_)_3_BH (3 equiv.), cat. acetic acid, 4-aminobutanol (1 equiv.), CH_2_Cl_2_, 0 °C to RT, 3 h, followed by: HF-pyridine (4 equiv.), THF, 0 °C, overnight.^8^ (f) Acid (1 equiv.), SOCl_2_ (1.1 equiv.), DMF (5 mol%), 2-MeTHF, 80 °C, 20 min.^9^ (g) Acyl chloride (1 equiv.), 1,6-hexanediol (3 equiv.), 2-MeTHF, 100 °C, 4 min.^9^ (h) 9 (1 equiv.), TEMPO (7 mol%), NaClO (2.3 equiv.), KBr (0.35 equiv.), 2-MeTHF, 2.3 min.^9^ (i) 3 (3 equiv.), 4-aminobutanol (1 equiv.), (CH_3_)_4_N(CH_3_CO_2_)_3_BH (3 equiv.), methanol, 2-MeTHF and NMP, 16 min.^9^ (j) X = Br, acid (1 equiv.), 6-bromo-1-hexanol (0.98 equiv.), TsOH (10 mol%), toluene, reflux, 16 h. (k) X = OH, acid (1 equiv.), 1,6-hexanediol (1 equiv.), EDC HCl (1.7 equiv.), DMAP (1.2 equiv.) CH_2_Cl_2_, 16 h. (l) 2 (1 equiv.), pyridine N-oxide (6 equiv.), NaOAc (2 equiv.), *n*-propyl acetate, reflux, 16 h. (m) 9 (1 equiv.), PIDA (1.1 equiv.), TEMPO (10 mol%), 10 : 1 heptane : CH_2_Cl_2_, 3–16 h. (n) Na_2_S_2_O_5_ (0.6 equiv.). (o) 4 (2.3 equiv.), 4-amino-1-butanol (1 equiv.), NEt_3_ (2.4 equiv.), NaBH(OAc)_3_ (4.3 equiv.), 2-MeTHF, 16 h.

There are select few syntheses of ALC-0315 1 that are reported (Scheme 1).^7–10^ However, they all have significant drawbacks from a process chemistry perspective, using skin sensitizing reagents, chlorinated solvents and having a low control on impurity management requires the use of column chromatography purification at multiple steps. To overcome these challenges and allow for a more sustainable and scalable synthesis, it was envisioned that the desired lipid could be obtained *via* a fatty aldehyde bisulfite adduct intermediate 4 of the analogous aldehyde 3 which would itself act as a purification handle in the synthesis. Additionally, the bisulfite adduct can be used directly in the next reductive amination step to produce 1.

**Chart 1:**
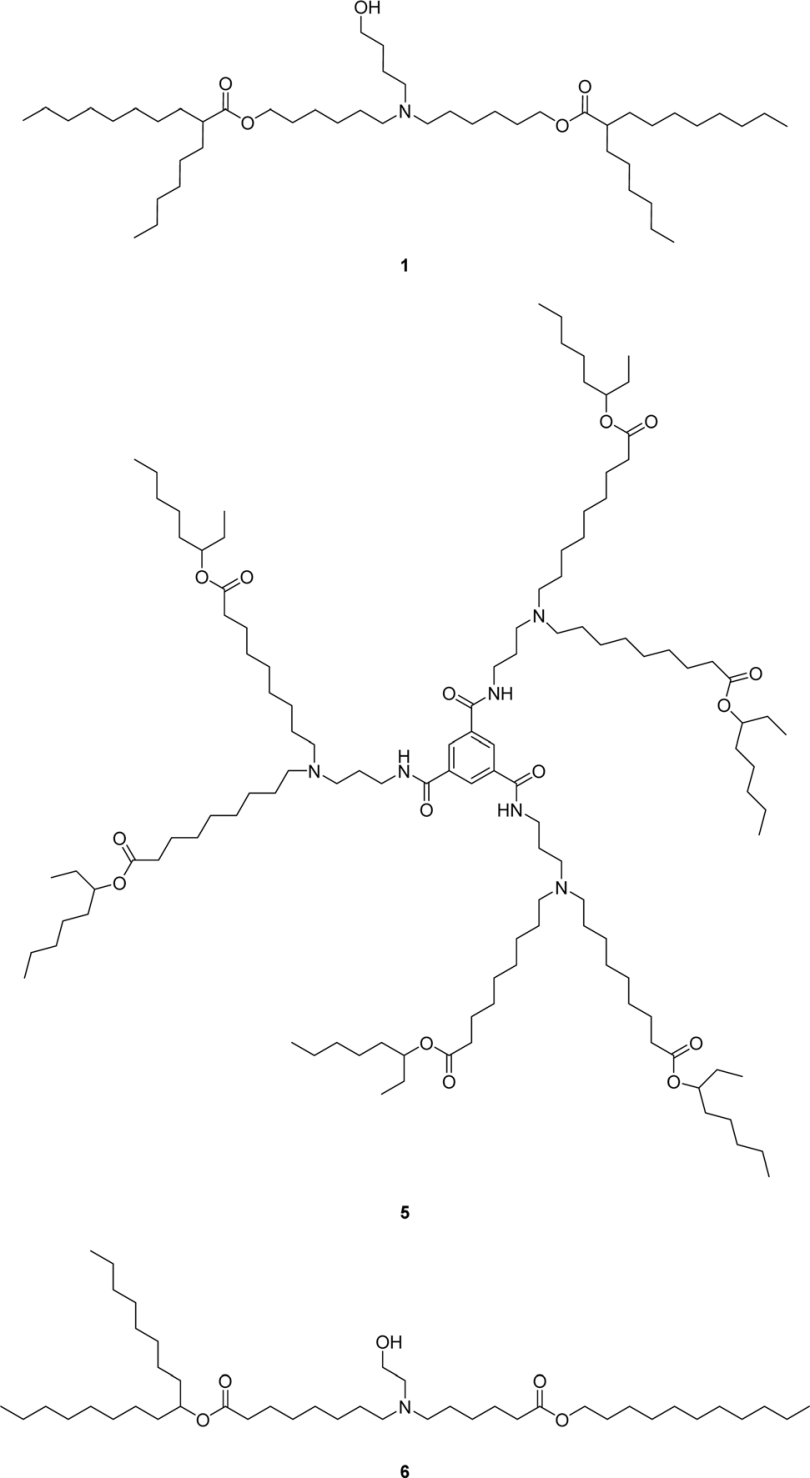
Lipids ALC-0315 1, FTT5 5 and SM-102 6.

To generate the required aldehyde 3, an acid-catalyzed esterification using the non-symmetrical 6-bromo-1-hexanol could be employed to avoid bis-ester formation. Whereas, prior reports relied on non-selective esterification of symmetrical diols, which typically requires the use of chromatographic separation of the undesired bis-ester byproduct or the use of a large molar excess of diol starting material relative to the carboxylic acid in order to disfavor bis-ester formation. The obtained bromo-ester 2 could then undergo a Ganem type oxidation to furnish the aldehyde 3.^11,12^ This would allow the aldehyde 3 to be purified *via* bisulfite adduct formation. Alternatively, the direct alkylation of the amine with the bromo-ester 2 to form ALC-0315 1 is known in the literature but produces the quaternary ammonium salt as a side-product which is not easily removed or controlled (Scheme 1, conditions b).^7^ Finally, a modification of the known reductive amination would provide ALC-0315 1 by direct reaction of the fatty aldehyde bisulfite adduct 4 in 2-MeTHF as an alternative to the commonly used CH_2_Cl_2_, increasing the selectivity without needing to add solubilizing groups such as silyl ethers (Scheme 1, conditions e)^8^ which can increase step count as well as unit operations dramatically. These modifications greatly improve the yield of the reductive amination to those reported in the literature.^7–10^ The strategy of purification *via* solid fatty aldehyde bisulfite adduct followed by reductive amination directly with the bisulfite adduct lends itself well to a large range of ionizable lipids due to structural similarities (Chart 1) and, therefore, retrosynthetic approaches. Select examples of other ionizable lipids (FTT5 5,^13^ a lipid-like compound which has found *in vivo* success, and SM-102 6 from Moderna's mRNA-1273 COVID-19 vaccine) were synthesized to further demonstrate the utility of this synthetic strategy.

To the best of our knowledge, solid fatty aldehyde bisulfite adduct intermediates have not been previously reported in the field of ionizable lipid synthesis nor used in subsequent reactions directly.

## Results

The initial step in the synthesis of ALC-0315 1 being an esterification allows for a variety of potential reaction conditions to be utilized. While carbodiimides are commonly used to couple an alcohol to a carboxylic acid for this step, an acid-catalyzed esterification can be envisioned to avoid skin sensitizers.^14–17^ Using standard Dean–Stark azeotropic conditions the bromo-ester 2 is generated in good yield (96%) and purity (>95% by HPLC-CAD).

With the bromo-ester 2 in hand, subsequent oxidation to the aldehyde was developed, which was adapted from an analogous oxidation (see ESI† for more details).^12^ Optimization of this reaction (Table S3†) culminates in bromide 2 being dosed into a refluxing solution of pyridine N-oxide, NaOAc, and *n*-propyl acetate over 4 hours to generate aldehyde 3 in good yield (88% NMR yield).

With general conditions for the alkyl bromide oxidation to an aldehyde, purification of the crude aldehyde 3*via* bisulfite adduct is seen as a means of not only overcoming impurities^18^ generated in the developed oxidation but as a potentially more appropriate starting material for the final reductive amination to generate 1.^19–21^ This would also act as a more suitable hold point when compared to typical aldehyde stability.^22^

Initial attempts to purify the crude aldehyde 3 involved dissolution of the crude oil in EtOH. This solution is then subjected to an aqueous solution of sodium metabisulfite dropwise. The bisulfite adduct 4 forms within 30 minutes (monitored by ^1^H NMR) but gives a sticky, gummy solid material which is not suitable for filtration. Aqueous extraction of the adduct is not feasible due to limited solubility.

Removal of the water that is introduced during bisulfite adduct formation *via* azeotropic distillation with toluene gives a more workable solid after addition of EtOH, but unfortunately is still unsuitable for filtration due to clogging.

Utilizing acetone as a bisulfite adduct anti-solvent has been reported previously^23^ and gives a well filtering mixture of 4, however a significant portion of the free aldehyde product is lost to the filtrates resulting in a decreased yield of the isolated solid adduct. It is proposed that a small equilibrium of the bisulfite adduct to the free aldehyde and bisulfite anion can cause the degradation of the adduct over time as acetone can also form a bisulfite adduct with the *in situ* generated bisulfite anion. Therefore, alternative anti-solvents were evaluated to avoid loss of the bisulfite adduct and to further improve the purification.

The results of a stability test of the adduct 4 indicate complete degradation of adduct and loss of product in acetone at extended contact times. Changing from acetone to ethyl formate improves the stability of the bisulfite adduct, as determined by ^1^H NMR (Fig. S1†), while still enabling filtration. This is likely due to the similar size and polarity of ethyl formate, while not reacting with the *in situ* generated bisulfite anions as acetone does. Additionally, spiking experiments also indicate that water does not have an impact on bisulfite stability (mechanistic detail Scheme S3†).

Upon addition of ethyl formate to the solid bisulfite material, a brown solution is obtained along with an easily filterable light-tan colored solid giving 23.86 g (73% yield) of 4 as a light-brown to tan solid from the starting bromo-ester 2. This tan solid is noted to be hygroscopic as before, and can be stored either under nitrogen, or in a desiccator. A significant amount of semi-solids are found to have collected on the underside of the filter paper, slowing filtration. This is likely due to the relatively low boiling point of ethyl formate and the low pressure experienced after passing through the filter. A simple change to DMC negates this issue and allows for a more rapidly filtering mixture.

The isolated adduct 4 is readily converted to the free aldehyde *via* washing with 10% sodium carbonate solution and extracting into EtOAc. The free aldehyde product is obtained with good purity (85–88%, HPLC-CAD) and can be used in the final reductive amination step. With the free aldehyde in hand from 4, the reductive amination to generate ALC-0315 1 is preformed *via* a dosed addition of the free aldehyde with portion-wise addition of the reducing agent, NaBH(OAc)_3_. Portion-wise addition of the reducing agent is needed to overcome process limitations.^8^ These conditions give purity of ALC-0315 1 in the isolated crude oil of 68% as analyzed by HPLC-CAD. Alternatively, the reductive amination can be performed directly from the isolated solid bisulfite adduct 4 (Scheme 1, conditions o) to give an improved crude purity (86% HPLC-CAD), while also allowing 2-MeTHF to replace CH_2_Cl_2_ as the solvent. Column chromatography gives purified ALC-0315 1 as a clear slightly yellow oil (53% isolated yield, 94.9% HPLC-CAD). This represents a 37% overall yield from the starting hexyldecanoic acid.

**Chart 2:**
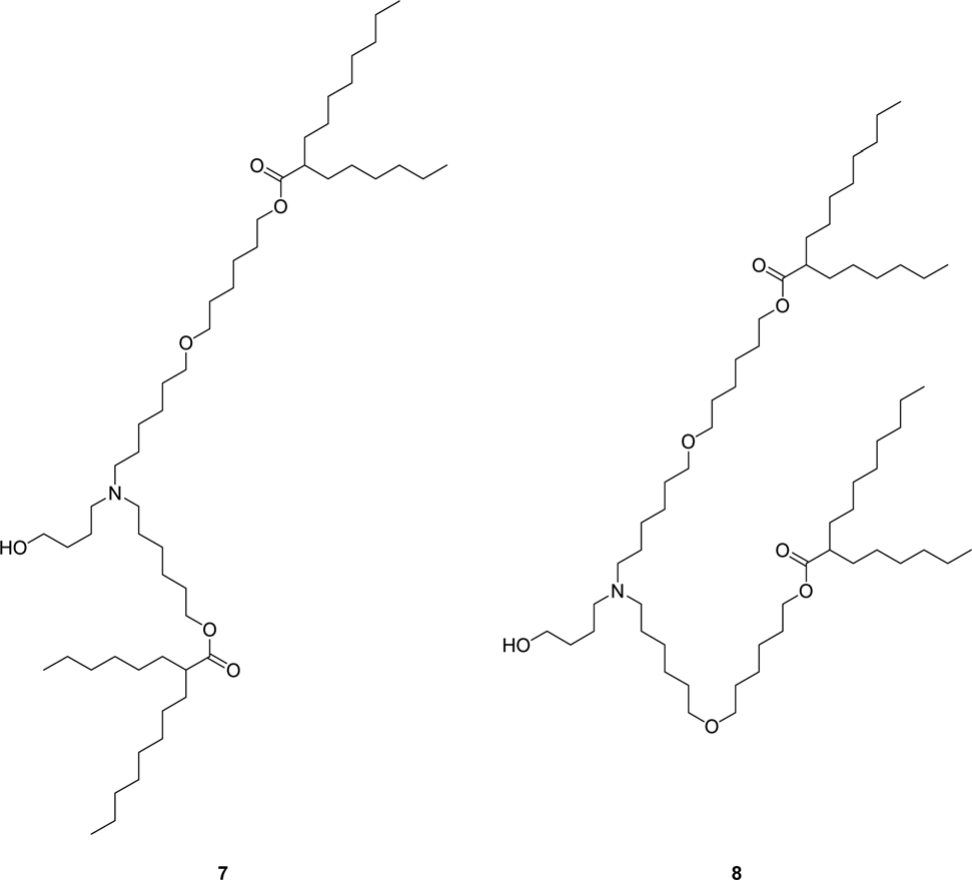
Structurally similar ALC-0315 impurities.

The main impurities in the final isolated ALC-0315 1 are two structurally related ether impurities, 7 and 8 (Chart 2), which stems from an ether impurity present in the 6-bromo-1-hexanol starting material. This ether impurity in the esterification starting material culminates in two structurally similar compounds to ALC-0315 which are not easily separated *via* column chromatography. Therefore, an alternative synthetic route was evaluated, avoiding 6-bromo-1-hexanol which contains the impurity.

**Fig. 1:**
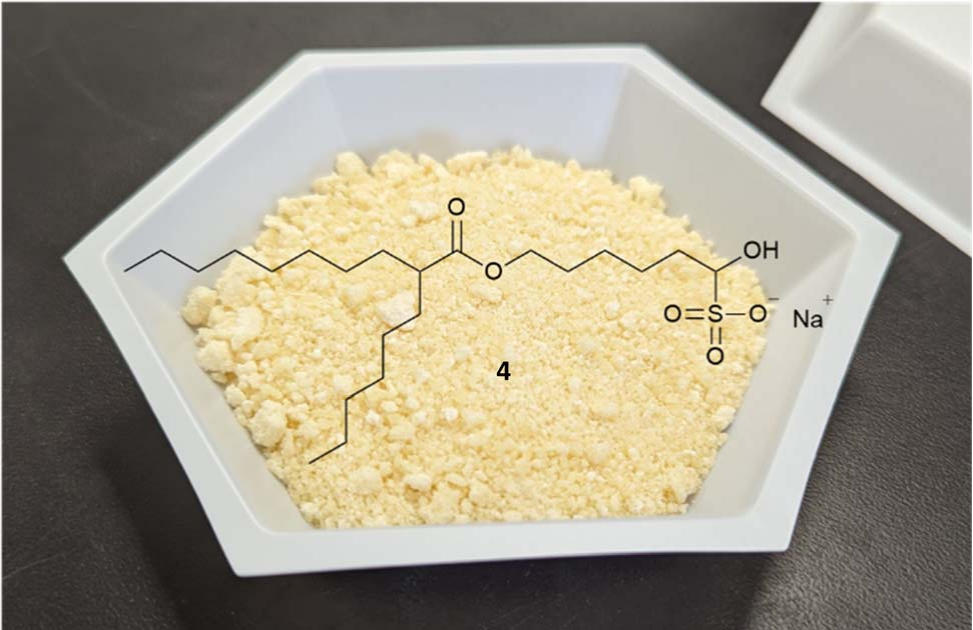
Photo of solid fatty bisulfite adduct 4 produced from 9.

A straightforward change of the first two steps eliminated the dependence on the problematic starting material and avoids the structurally similar impurities, 7 and 8, albeit with the introduction of a coupling reagent. An initial EDC coupling of hexyldecanoic acid and 1,6-hexandiol gives the mono-esterified diol 9 in high yield and good purity (94%, 88.0% HPLC-CAD), with the main impurity being the bis-ester byproduct (9.5%), which can be easily removed in a later step during purification of the aldehyde bisulfite-adduct. Alcohol-ester 9 is then oxidized to aldehyde 3 with PIDA in the presence of cat. TEMPO.^24–26^ The crude aldehyde 3 is then subjected to the general bisulfite adduct purification conditions. This gives crystalline off-white solid which filters rapidly to give the purified product as the sodium bisulfite adduct 4 (Fig. 1) in a 90% yield (83% purity by HPLC-CAD) from 9, and overall yield of 85% from hexyldecanoic acid. This solid isolate eliminates the need for a chromatographic purification prior to its use in the following step, significantly reducing waste that is typically generated in such chromatographic operations (Table S4†). The adduct 4, as before, is subjected to the improved reductive amination conditions and column purified to give ALC-0315 1 in a 64% yield for the final reductive amination step and 54% overall yield of ALC-0315 1 from the starting material, hexyldecanoic acid. This yield is an improvement when compared to other literature preparations of ALC-0315 1 (20–47%, Scheme 1)^7–9^ as well as patented procedures (50%).^10^ The culmination of the process yields ALC-0315 1 with a purity of 97.3% as analyzed by HPLC-CAD with no structurally similar impurities, 7 or 8, present. This material was subsequently used in a formulation preparation, encapsulating mRNA producing LNPs which match the physiochemical properties of formulation batches that utilize commercially available ALC-0315 (Table S6†).

With the ability to synthesize ALC-0315 1 in this manner, FTT5 5, a proven lipid *in vivo*,^13^ and SM-102 6 were the next targets for evaluating the route's feasibility across multiple lipids.

**Scheme 2:**
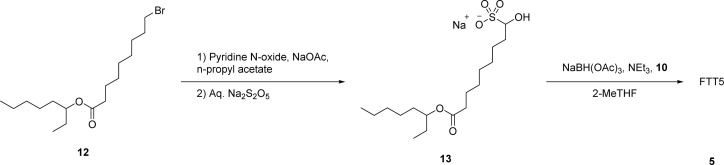
Synthetic route through solid bisulfite adduct 13 to lipid FTT5 5.

**Fig. 2:**
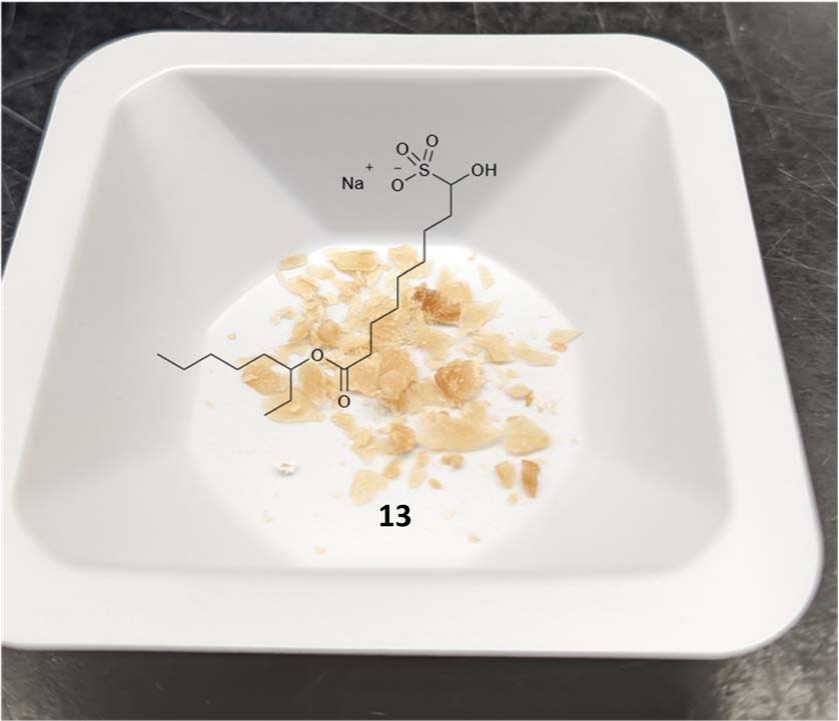
Photo of solid fatty bisulfite adduct 13.

Preparation of the bisulfite adduct intermediate 13 of FTT5 5 follows analogously to 4 (Schemes 2 and S1† for full route) and gives a well behaving flaky tan solid (Fig. 2). This, again, eliminates the need for a chromatographic separation of the aldehyde intermediate. Direct reaction of amine 10 with bisulfite adduct 13 gives FTT5 5 (26%), closely matching the literature yield (27%).^13^ The inclusion of IPA in the reaction is required to solubilize the amine 10, otherwise a very slow reaction is observed.

**Scheme 3:**
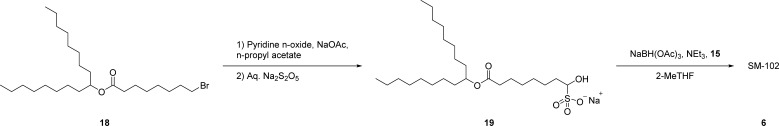
Synthetic route through solid bisulfite adduct 19 to lipid SM-102 6.

**Fig. 3:**
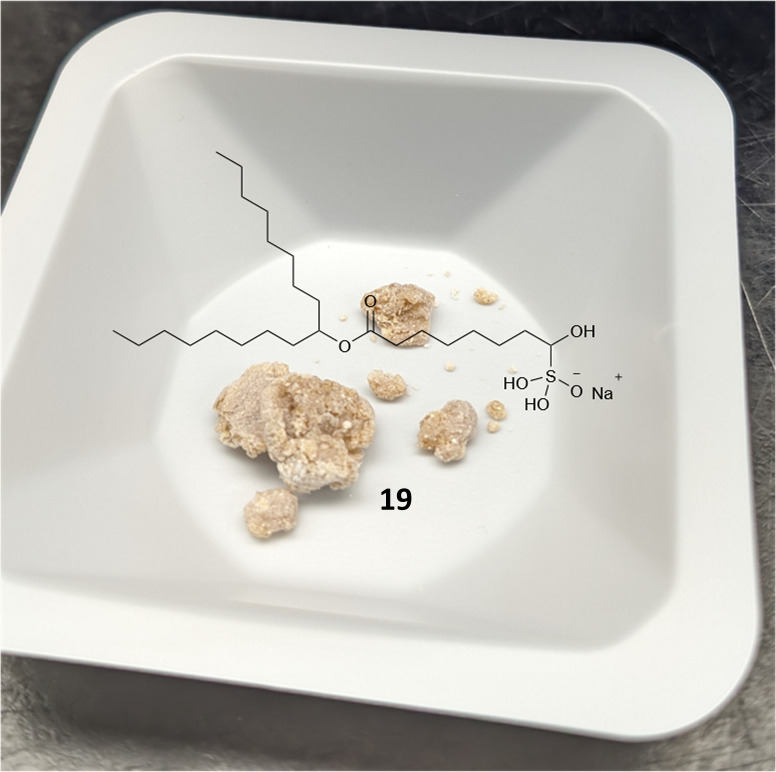
Photo of solid fatty bisulfite adduct 19.

The synthetic route to SM-102 6 proceeds through bisulfite adduct 19, after oxidation of bromo-ester 18 (Schemes 3 and S2† for full route). Isolation of the bisulfite adduct 19 gives a well behaving off-white to tan solid (Fig. 3). After direct reaction of bisulfite adduct 19 with amine 15 and column purification, SM-102 6 is obtained in good yield and purity (67% yield, 96.0% purity by HPLC-CAD). As with ALC-0315 1 and FTT5 5, SM-102 6 is readily prepared *via* a solid aldehyde bisulfite adduct intermediate, enabling the elimination of chromatographic purification of the aldehyde intermediate, as well as the use of the bisulfite adduct 19 directly in the final step to produce the lipid.

## Experimental

### Synthetic procedures

#### General procedure for synthesis of bromo-esters from bromo-alcohols

To a 3-neck RBF with attached Dean–Stark (open at top) and nitrogen inlet is added the acid (1 eq.), followed by toluene (5 V), bromo-alcohol (0.98 eq.), and *p*-TsA (0.1 eq.). This is brought up to a reflux with a very slight nitrogen flow. Upon collection of distillates in the Dean–Stark, the Dean–Stark is filled to just below overflow with toluene and the reaction is allowed to reflux for 16 hours or until the reaction is complete *via*^1^H NMR before it is cooled to room temperature. The reaction mixture is transferred to a separatory funnel and the RBF is washed with 5 V sat. NaHCO_3_. This is transferred into the separatory funnel, shaken, and allowed to separate. The organic layer is separated, dried (Na_2_SO_4_) and concentrated to give a slightly yellow to light brown oil.

#### General procedure for synthesis of bromo-esters from bromo-acids

To a 3-neck RBF with attached Dean–Stark (open at top) and nitrogen inlet is added the bromo-acid (0.98 eq.), followed by toluene (5 V), alcohol (1 eq.), and *p*-TsA (0.1 eq.). This is brought up to a reflux with a very slight nitrogen flow. Upon collection of distillates in the Dean–Stark, the Dean–Stark is filled to just below overflow with toluene and the reaction is allowed to reflux for 16 hours or until the reaction is complete *via*^1^H NMR before it is cooled to room temperature. The reaction mixture is transferred to a separatory funnel and the RBF is washed with 5 V sat. NaHCO_3_. This is transferred into the separatory funnel, shaken, and allowed to separate. The organic layer is separated, dried (Na_2_SO_4_) and concentrated to give a slightly yellow to light brown oil.

#### General procedure for synthesis of fatty aldehyde bisulfite adducts

To a RBF with attached condenser is added pyridine N-oxide (6.0 eq.), *n*-propyl acetate (9 V), and sodium acetate (2.0 eq.). This is brought to a reflux before the bromo-ester is added, as a solution in *n*-propyl acetate (1 V), to the refluxing solution over 4 hours. After addition is complete the reaction is allowed to reflux for a further 16 hours before cooling to room temperature. To the cooled mixture is added HPW (10 V). This is shaken, separated and organic is washed with sat. NaHCO_3_ (10 V) before drying over Na_2_SO_4_ and filtering. To the *n*-propyl acetate solution at 35 °C is added sodium metabisulfite as a solution in water (0.45 g mL^−1^) dropwise. This is allowed to stir at 35 °C for 30 minutes before concentrating *in vacuo*. After concentrating an additional 10 V *n*-propyl acetate is charged and concentrated giving a solid material. To the solid is added ethyl formate or DMC (5 V) and the mixture is stirred at room temperature until all the solid material has formed a white to tan solid suspension in the mixture. This mixture is filtered, washed with ethyl formate or DMC (2 V) and the solids are collected and dried in a vacuum oven for 16 hours with a slight nitrogen bleed before weighing the collected bisulfite adduct.

#### General procedure for synthesis of ionizable lipids *via* reductive amination of fatty aldehyde bisulfite adducts

To a RBF with attached nitrogen inlet is added bisulfite adduct (2.3 eq.), and 2-MeTHF (10 V to the adduct) at RT. This is allowed to stir until a homogeneous mixture is observed. NEt_3_ (2.4 eq.) is added to the reaction followed by the reactive amine (1 eq.) and then NaBH(OAc)_3_ (4.3 eq.). The reaction is stirred for 16 h at RT before the reaction solution is washed with sat. Na_2_CO_3_ (10 V to adduct). The organic layer is dried (Na_2_SO_4_), filtered, and concentrated before being purified *via* column chromatography (SiO_2_).

## Conclusions

A general route has been developed for multiple commonly used ionizable lipids which have been prepared through solid fatty aldehyde bisulfite adduct intermediates, eliminating the need for column purification of intermediates in these multi-step syntheses. Both ALC-0315 1 and SM-102 6, two ionizable lipids being used in COVID-19 vaccine drug products, as well as FTT5 5, a relatively recent lipid-like compound which has seen *in vivo* success, have been prepared by employing fatty aldehyde bisulfite adducts, which allows for easy isolation of the resulting solid intermediates. Additionally, the bisulfite adducts can be used directly in subsequent reductive amination reactions, in which they performed equally or better than the free aldehyde while also eliminating the need for CH_2_Cl_2_ in the reaction. We believe this to be the first reported isolation of solid fatty aldehyde bisulfite adduct intermediates and their use in the production of ionizable lipids.

The production of ALC-0315 1*via* a solid fatty aldehyde bisulfite adduct intermediate enabled an improved purification process to the typical column chromatography, improved yield when compared to other syntheses, and was found to perform equally when formulated with mRNA as other commercial sources of ALC-0315. Isolation of solid fatty aldehyde intermediates *via* bisulfite adduct formation and filtration is viewed as a general approach to improve synthesis of other lipids and the isolation and purification of other fatty alkyl aldehyde intermediates.

## Data availability

The data supporting this article have been included as part of the ESI.†

## Conflicts of interest

There are no conflicts to declare.

## Supplementary Material

RA-014-D4RA05189K-s001
