# Supplementary material for: Fatty aldehyde bisulfite adducts as a purification handle in ionizable lipid synthesis
Source: RSC Adv. 2024 Aug 19;14(36):26233–8. doi: 10.1039/d4ra05189k (PMC11332587; doi:10.1039/d4ra05189k)
Supplement: RA-014-D4RA05189K-s001 [file RA-014-D4RA05189K-s001.pdf]

Supplementary Information (SI) for RSC Advances.
This journal is © The Royal Society of Chemistry 2024

Fatty Aldehyde Bisulfite Adducts as a Purification Handle in Ionizable Lipid Synthesis

Graham Atwood, Sona Purbiya, Cassandra Reid, Brandon Smith, Kuljit Kaur, Drew Wicks, Peter Gaudet, K. Cory MacLeod and Jean-Francois Vincent-Rocan*

[1] *Biovectra Inc., 11 Aviation Avenue, Charlottetown, Prince Edward Island C1E 0A1, Canada,*

**Correspondence and requests for materials should be addressed to JF-VR. (email: jvincent-rocan@biovectra.com)*

List of Contents

1. General Information and Materials 3
2. Experimental Details 5
    2.1. Procedures for the Synthesis of Compounds 6
    2.2. Optimization of Bromide Oxidation Reaction Conditions........17
    2.3. Aldehyde Purification Metrics........21
    2.4. Formulation Results 21
    2.5. Bisulfite Adduct Stability Study 21
3.1. ^{1}H NMR Spectra 23
4.1. ^{13}C NMR Spectra 37
5.1. HPLC-CAD Chromatograms........39

1. General Information and Materials

All reactions were performed under a positive pressure of nitrogen or argon unless noted otherwise. All solvents were used as is from their respective suppliers. All column purified materials were purified on a BUCHI Pure Chromatography System with UV and/or ELSD detectors. Columns packed with 25 um spherical silica gel. The ^{1}H NMR (300 MHz) spectra as for solution in $CDCl_3$ or DMSO-d_6 or MeOH-d_4 were recorded on a Varian Mercury 400 MHz spectrometer. The chemical shifts (δ) are expressed in ppm downfield from internal TMS (δ = 0.00), or $CDCl_3$ (δ = 7.26), or DMSO-d_6 (δ = 2.50), or MeOH-d_4 (δ = 3.31) and coupling constants (*J*) are reported in hertz (Hz). The following abbreviations were used to explain the multiplicities: s = singlet, d = doublet, t = triplet, q = quartet, m = multiplet, br = broad. Tetramethyl pyrazine or $DMSO_2$ used as ^{1}H NMR standards.

Commercially available chemicals were obtained from Aldrich Chemical Co., Oakwood Chemical, TCI, combi-blocks and used as received unless otherwise noted.

Where applicable procedures used in the preparation of previously prepared compounds are referenced after the title of the given preparation. Where applicable NMR spectra of previously prepared compounds are referenced after the title of the spectra.

HPLC-CAD analysis preformed on Waters Acquity Arc system with a Thermo Corona Veo RS charged aerosol detector with a Phenomenex Luna C18(2) 100 Å, 150 mm x 3 mm ID, 5 µm column. (Column temperature = 50 °C, sample temperature = 5 °C). Mass analysis preformed on a Waters Acquity QDa.

**CAD Evaporative Temperature**	40°C
**Power Function**	1.00
**Data Collection Rate**	5 Hz
**Filter**	3.6
**Sample Diluent**	Anhydrous Ethanol
**Sample Concentration**	1 mg/mL
**Injection Volume**	5 µL
**Mobile Phase A**	10mM Ammonium Formate in Milli-Q Water + 0.1% Formic Acid
**Mobile Phase B**	10mM Ammonium Formate in (62:33:5 v/v) IPA: ACN: Water + 0.1% Formic Acid
**Mobile Phase C**	IPA
**Mobile Phase D**	ACN
**Sample Manager Purge/Wash**	ACN
**Seal Wash**	20% ACN
**Gradient**	(see below)

Gradient

Time (min)	Flow (mL/min)	% Mobile A	% Mobile B	% Mobile C	% Mobile D	Curve
Initial	0.800	30.0	70.0	0.0	0.0	
2.00	0.800	30.0	70.0	0.0	0.0	6
15.00	0.800	0.0	100.0	0.0	0.0	6
25.00	0.800	0.0	100.0	0.0	0.0	6
28.00	0.800	0.0	0.0	100.0	0.0	6
33.00	0.800	0.0	0.0	100.0	0.0	6
36.00	0.800	30.0	70.0	0.0	0.0	6
40.00	0.800	30.0	70.0	0.0	0.0	6
45.00	0.800	0.0	0.0	0.0	100.0	11
50.00	0.800	0.0	0.0	0.0	100.0	11

Table S1 HPLC-CAD method parameters

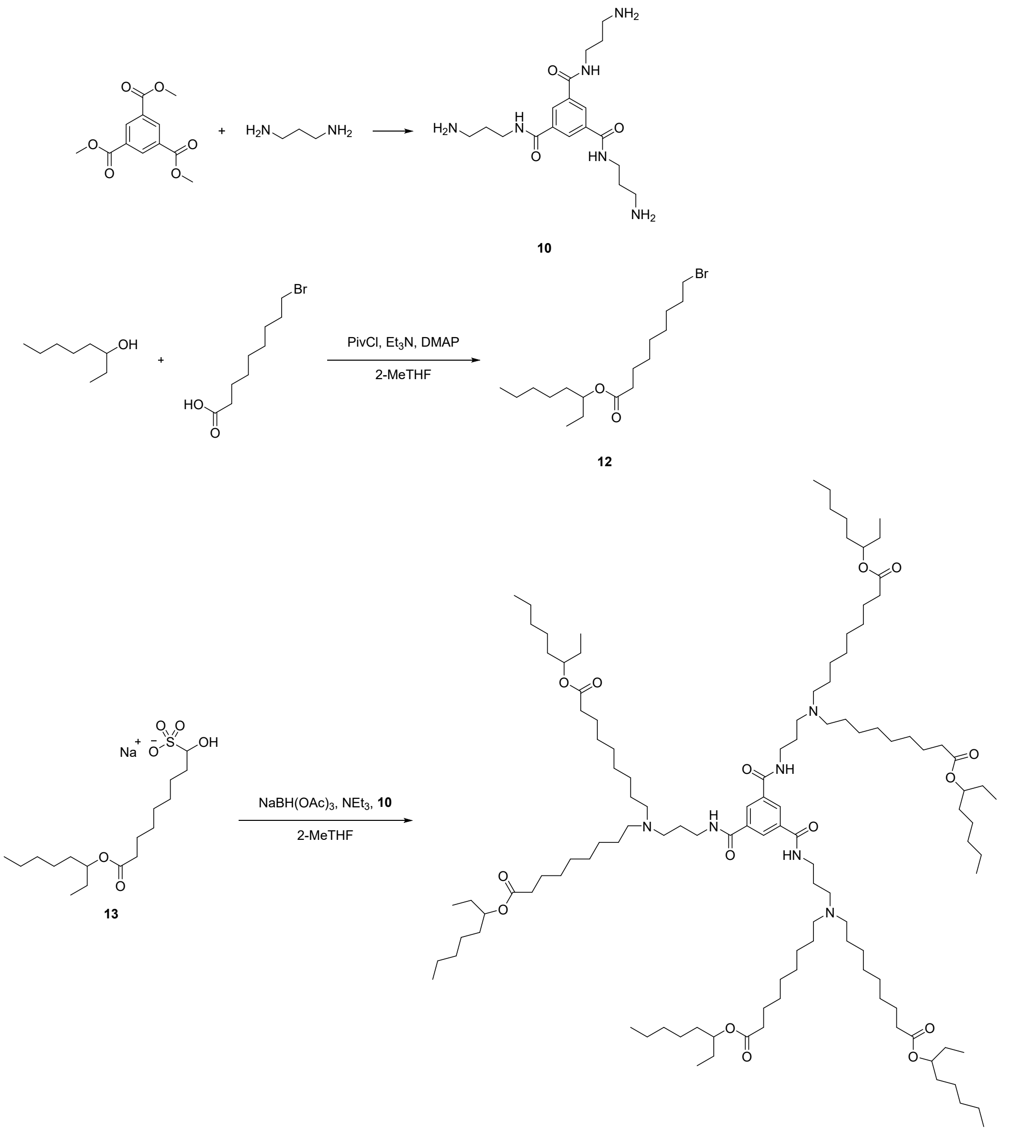

Scheme S1 Synthetic route to lipid FTT5 **5**

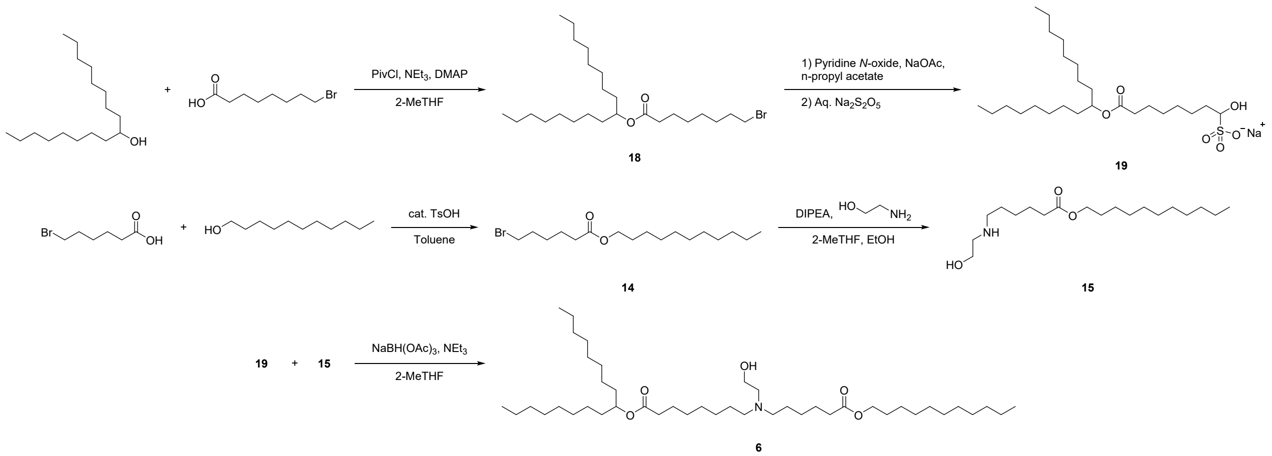

Scheme S2 Synthetic route to lipid SM-102 **6**

2. Experimental Details

2.1. Procedures for the Synthesis of Compounds

Synthesis of ALC-0315 1[1–3]

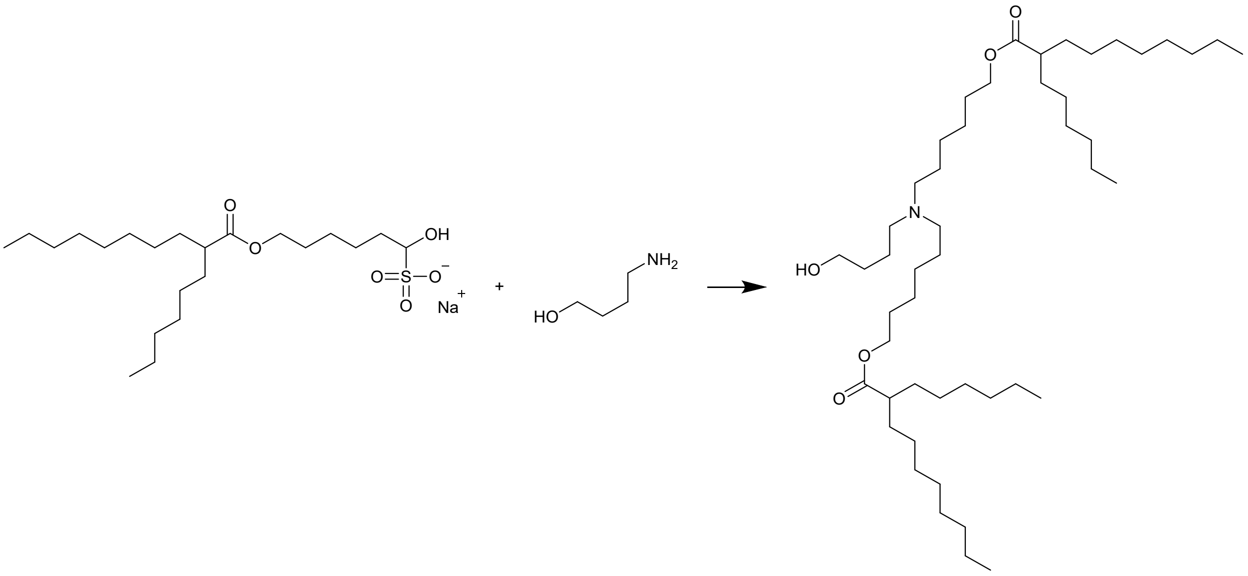

Compound was prepared via the general procedure for synthesis of ionizable lipids via reductive amination of fatty aldehyde bisulfite adducts. Flash purification (2% NEt_3 CH_2Cl_2 : 2% NEt_3 EtOAc, 1:0 → 0:1) gives ALC-0315 **1** as a slightly yellow oil (18.6845 g, 64%)

^{1}H NMR (400 MHz, $CDCl_3$) δ 6.84 – 6.33 (bs, 1H), 4.06 (t, J=6.65 Hz, 4H), 3.52-3.58 (m, 2H), 2.39-2.48 (m, 6H), 2.26-2.36 (m, 2H), 1.18-1.72 (m, 69H), 0.88 (t, J=6.65 Hz, 12H)

Purity: 94.9% (HPLC-CAD) (From 6-bromohexyl 2-hexyldecanoate **2)**

Purity: 97.3% (HPLC-CAD) (From 6-hydroxyhexyl 2-hexyldecanoate **9)**

Synthesis of 6-bromohexyl 2-hexyldecanoate 2

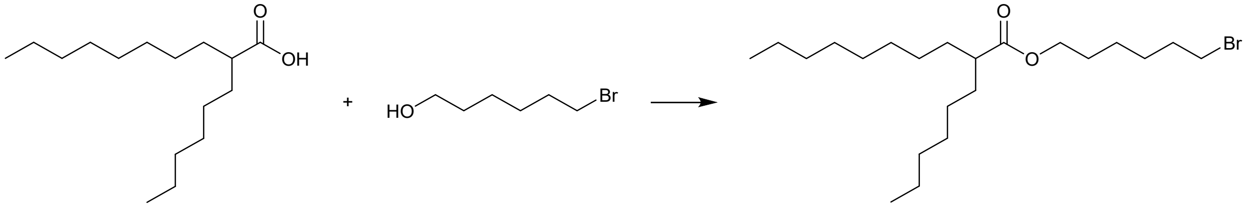

Compound was prepared using the general procedure for synthesis of bromo-esters from bromo-alcohols. (157.02 g, 96%)

^{1}H NMR (400 MHz, $CDCl_3$) δ 4.08 (t, J = 6.6 Hz, 2H), 3.42 (t, J = 6.8 Hz, 2H), 2.32 (tt, J = 9.0, 5.3 Hz, 1H), 1.93 – 1.82 (quin, J = 7.14 Hz, 2H), 1.70 – 1.16 (m, 31H), 0.93 – 0.81 (m, 6H)

Synthesis of sodium 6-((2-hexyldecanoyl)oxy)-1-hydroxyhexane-1-sulfonate 4

From 6-bromohexyl 2-hexyldecanoate **2**:

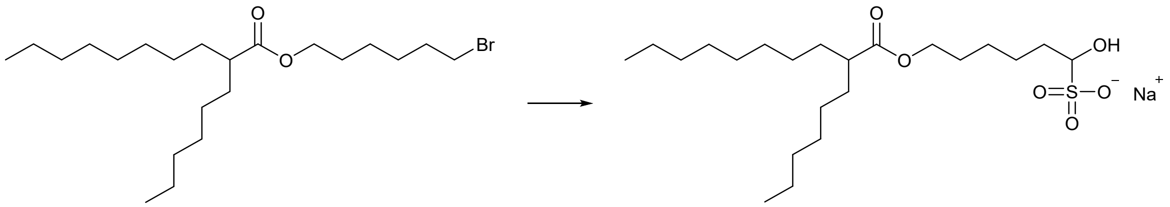

Compound was prepared via the general procedure for synthesis of fatty aldehyde bisulfite adducts. (23.86 g, 73%)

From 6-hydroxyhexyl 2-hexyldecanoate **9**:

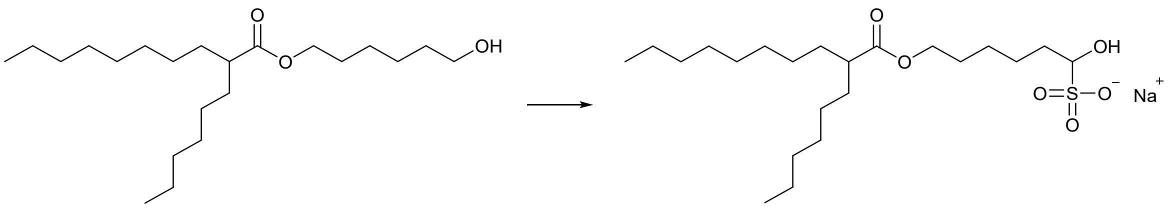

To a RBF is added the ester alcohol **9** (37.0194 g, 104.07 mmol) followed by heptane:CH_2Cl_2 (10:1, 555 mL). This is allowed to dissolve before PIDA (36.7879 g, 114.23 mmol) is added, followed by TEMPO (1.6237 g, 10.40 mmol) giving an orange mixture with white suspended solids. This is allowed to stir until no alcohol is present via ^{1}H NMR. Sat. $NaHCO_3$ (300 mL) is slowly added to the reaction to slow off-gassing. After addition, the biphasic mixture is stirred for 30 minutes before the organic layer is separated and the aqueous is reextracted with CH_2Cl_2 (250 mL x 2). All organic layers are combined and dried (Na_2SO_4) before concentration to give a crude red oil red oil (53.09 g). To the red oil is added n-propyl acetate (350 mL) and stirred at 35 °C until fully dissolved. To this is added sodium metabisulfite in HPW (12.1539g in 26 mL) dropwise. This is allowed to stir for 20 hours or until no aldehyde signal is noted by ^{1}H NMR. The solution is then concentrated *in vacuo* before n-propyl acetate (350 mL) is added and concentrated again giving a solid glass-like material. DMC (175 mL) is added to the solid and is stirred/shaken until a solid suspension is noted. This is filtered to give a slightly yellow solid. The solids are washed with DMC (70 mL) before transferring to a drying dish and drying via vacuum oven for 16 hours. This gives the title compound as a slightly yellow solid (42.79 g, 90%).

^{1}H NMR (400 MHz, DMSO-d_6) δ 5.24 (br d, J = 5.1 Hz, 1H), 3.99 (t, J = 6.5 Hz, 2H), 3.84 – 3.73 (m, 1H), 2.27 (tt, J = 9.2, 4.9 Hz, 1H), 1.81 – 1.65 (m, 1H), 1.59 – 1.07 (m, 33H), 0.84 (t, J = 6.1 Hz, 6H)

^{13}C NMR (101 MHz, DMSO-d_6) δ 175.30, 82.81, 63.47, 44.85, 31.88, 31.52, 31.19, 31.03, 28.73, 28.80, 28.73, 28.53, 28.47, 28.20 26.70, 25.35, 25.16, 22.03, 21.94, 13.83, 13.82

Synthesis of FTT5 5[4]

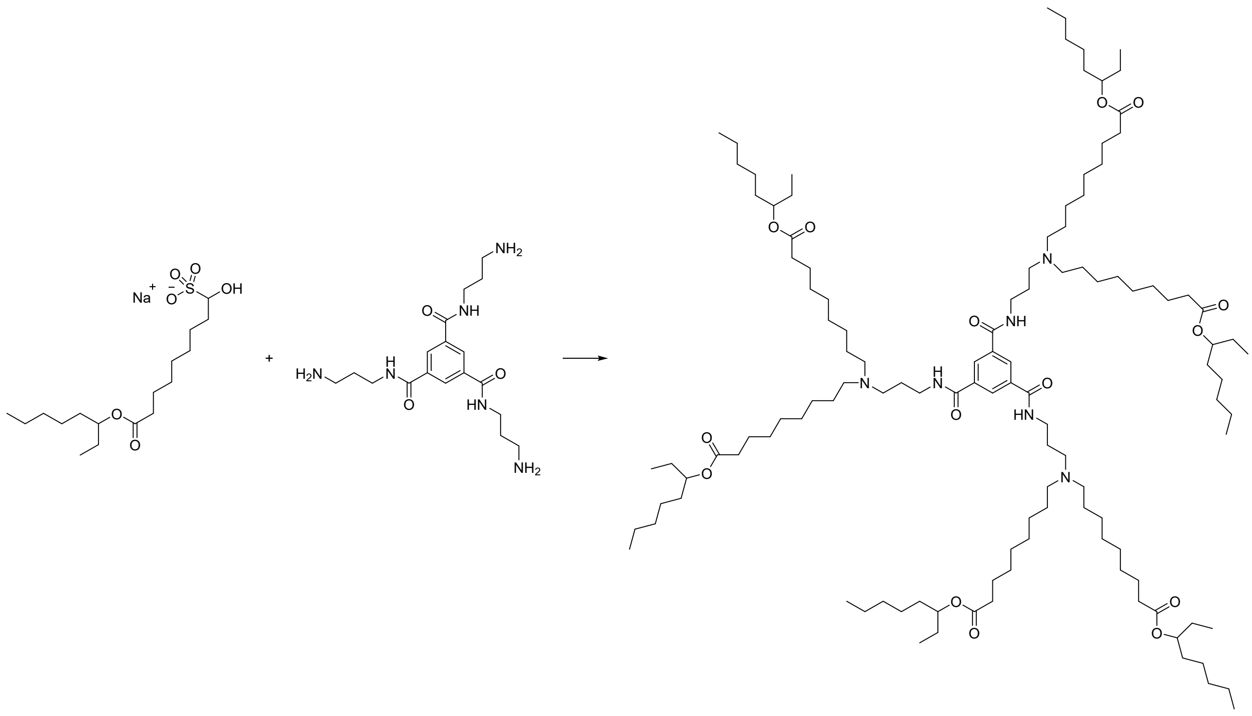

To an RBF is added the triamine **10** (1.0578 g, 2.7927 mmol), and IPA (70 mL). This is warmed to 35 °C before the adduct **13** (8.3641 g, 21.529 mmol) is added followed by $NaBH(OAc)_3$ (8.5695 g, 40.437 mmol), NEt_3 (2.24 g, 22.128 mmol) and finally 2-MeTHF (200 mL). This was allowed to stir at 35 °C for 96 hours before the content for the RBF is transferred to a separatory funnel and 10% Na_2CO_3 (70 mL) is added to the funnel along with EtOAc (70 mL). This is shaken, organic separated and aqueous is reextracted with CH_2Cl_2 (50 mL). The organic layers are combined, dried (Na_2SO_4), and concentrated before purifying via flash chromatography (CH_2Cl_2:Ultra 1:0 → 0:1, Ultra = 75/23/2 v/v/v CH_2Cl_2/MeOH/NH_4OH) to give FTT5 as a waxy yellow oil (1.39 g, 26%).

^{1}H NMR (400 MHz, $CDCl_3$) δ 8.94 – 8.03 (bd, 6H), 4.91 – 4.68 (m, 6H), 3.79 – 3.43 (m, 6H), 3.43 – 2.38 (m, 16H), 2.28 (br t, J = 7.2 Hz, 13H), 2.19 – 1.88 (m, 6H), 1.55 (m, 50H), 1.42 – 1.11 (m, 85H), 0.95 – 0.77 (m, 36H)

Synthesis of SM-102 6

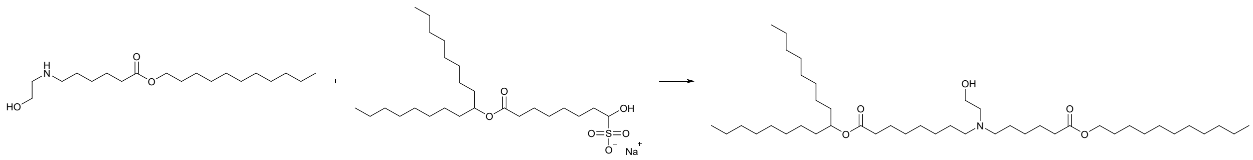

The compound was prepared using the general procedure for synthesis of ionizable lipids via reductive amination of fatty aldehyde bisulfite adducts. Flash purification (CH_2Cl_2 : 2% NEt_3 EtOAc, 1:0 → 0:1) gives SM-102 **6** as a slightly yellow oil (2.8771 g, 67%)

^{1}H NMR (400 MHz, $CDCl_3$) δ 4.85 (quin, J = 6.3 Hz, 1H), 4.03 (t, J = 6.8 Hz, 2H), 3.50 (t, J = 5.5 Hz, 2H), 3.18-2.81 (bs, 1H), 2.54 (t, J = 5.3 Hz, 2H), 2.48 – 2.35 (m, 4H), 2.27 (m, 4H), 1.68 – 1.37 (m, 14H), 1.36 – 1.13 (m, 46H), 0.85 (m, 8H)

Purity: 96.0% (HPLC-CAD)

Synthesis 6-hydroxyhexyl 2-hexyldecanoate 9[1,3]

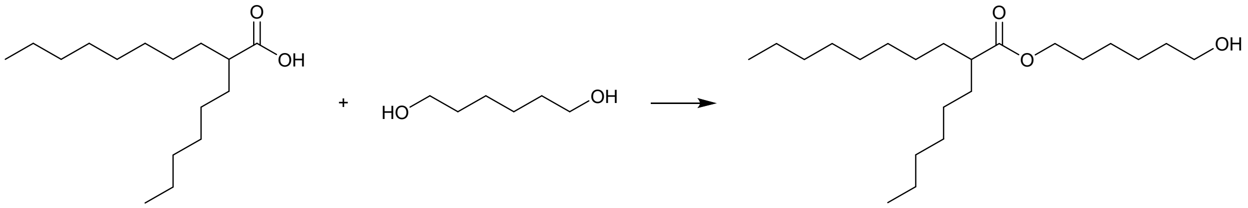

To an RBF with the 1,6-hexandiol (82.956 g, 116.99 mmol) is added CH_2Cl_2 (1.2 L). This is warmed to 35 °C and stirred until fully dissolution of the diol before DMAP (17.151g, 140.39 mmol), hexyldecanoic acid (30.000 g, 116.99 mmol), and finally EDC HCl (38.126 g, 198.88 mmol). This is allowed to stir at 35 °C for 16 hours or until all hexyldecanoic acid is consumed. The reaction is washed with HCl (400 mL, 1M), HPW (400 mL), and finally sat. $NaHCO_3$ (400 mL) before drying (Na_2SO_4) and concentrating to give the title compound as a clear colorless oil (39.0599 g, 94%)

^{1}H NMR (400 MHz, $CDCl_3$) δ 4.07 (t, J=6.45 Hz, 2H), 3.64 (q, J = 5.9 Hz, 2H), 2.36 – 2.24 (m, 1H), 1.70 – 1.12 (m, 34H), 0.88 (m, 6H)

Synthesis of N1,N3,N5-tris(3-aminopropyl)benzene-1,3,5-tricarboxamide 10[5]

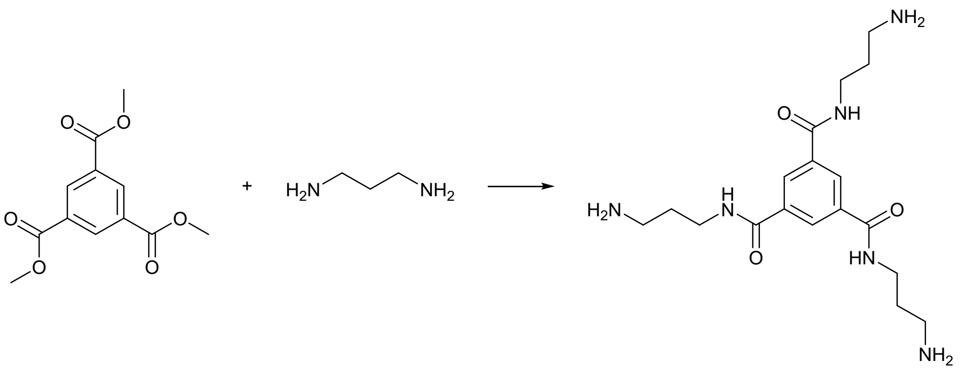

To an RBF is added propane-1,3-diamine (10.7085 g, 144.43mmol), and trimethyl benzene-1,3,5-tricarboxylate (3.0042 g, 14.50mmol). This is allowed to stir at room temperature for 24 hours before MeOH (100 ml) was added, and the contents of the reaction was concentrated *in vacuo*. This was repeated two further times before toluene (100 ml) is added, and the contents of the RBF is again concentrated to give the title compound as a white/clear solid (4.8789 g, 89%).

^{1}H NMR (400 MHz, MeOH-d_4) δ 8.39 (s, 3H), 3.48 (t, J = 6.8 Hz, 6H), 2.72 (t, J = 6.8 Hz, 6H), 1.78 (quin, J = 6.8 Hz, 6H)

Synthesis of octan-3-yl 9-bromononanoate 12[6]

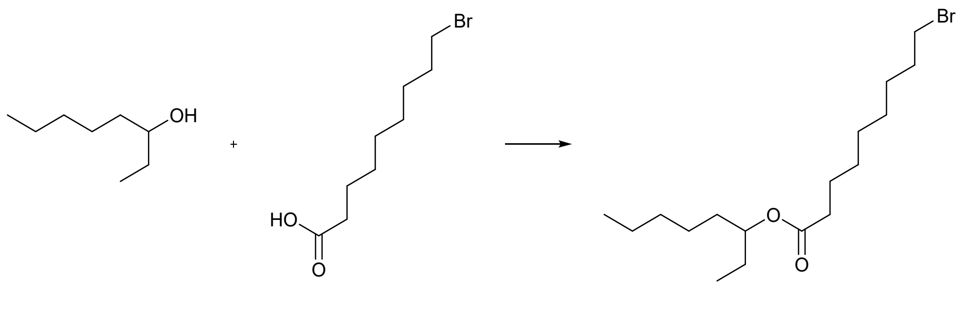

To an RBF is added the bromo-acid **12** (10.0834 g, 42.5217 mmol), 2-MeTHF (50 mL) and DMAP (258.3 mg, 2.116 mmol). This is allowed to stir and fully dissolve before the RBF is cooled in an ice bath. To the cooled solution is added NEt_3 (8.9614 g, 88.57 mmol), and then PivCl (5.2971g, 43.898 mmol) dropwise. This was allowed to stir for 30 minutes before 3-octanol as a solution in 2-MeTHF (5.772 g, 44.30 mmol in 150 mL) is added to the RBF slowly. The RBF is brought to RT and stirred for 23 hours before the RBF is transferred into a separatory funnel followed by sat. $NaHCO_3$ (50 mL) and EtOAc (40 mL). This was shaken, the organic layer separated before drying (Na_2SO_4) and concentrating to give the title compound as a slightly yellow oil. (16.57 g, 112%).

^{1}H NMR (400 MHz, $CDCl_3$) δ 4.77 (quin, J = 6.2 Hz, 1H), 3.35 (t, J = 6.8 Hz, 2H), 2.24 (t, J = 7.6 Hz, 2H), 1.80 (quin, J = 7.1 Hz, 2H), 1.64 – 1.13 (m, 20H), 0.89 – 0.76 (m, 6H)

Synthesis of sodium 1-hydroxy-9-(octan-3-yloxy)-9-oxononane-1-sulfonate 13

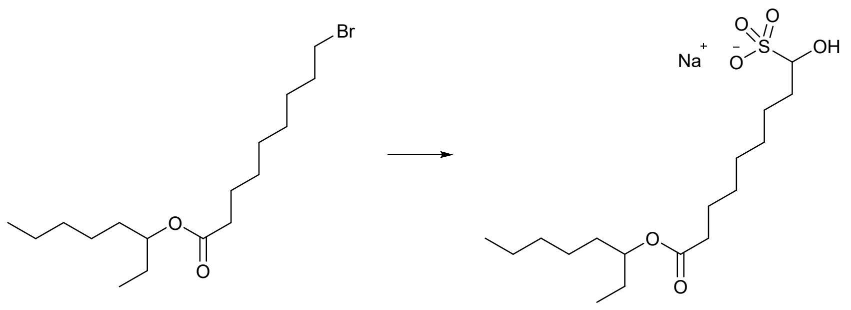

Compound was prepared using the general procedure for synthesis of fatty aldehyde bisulfite adducts. (9.67 g, 58%)

^{1}H NMR (400 MHz, DMSO-d_6) δ 5.19 – 5.00 (m, 1H), 4.77 – 4.66 (m, 1H), 3.74 (br d, J = 7.0 Hz, 1H), 2.26 (t, J = 7.0 Hz, 2H), 1.79 – 1.63 (m, 1H), 1.60 – 1.12 (m, 20H), 0.92 – 0.73 (m, 5H)

^{13}C NMR (101 MHz, DMSO-d_6) δ 172.60, 82.88, 74.25, 33.79, 32.98, 30.99, 28.88, 28.44, 26.49, 25.47, 24.66, 24.36, 21.91, 13.74, 9.40

Synthesis of undecyl 6-bromohexanoate 14[7]

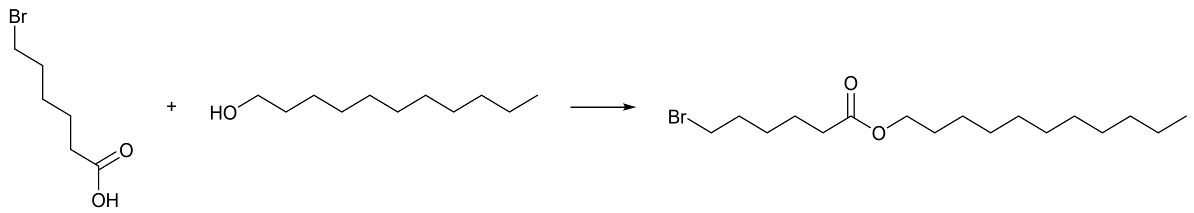

Compound was prepared using the general procedure for synthesis of bromo-esters from bromo-acids. (15.5819 g, 87%)

^{1}H NMR (400 MHz, $CDCl_3$) δ 4.06 (t, J = 6.5 Hz, 2H), 3.41 (t, J = 6.6 Hz, 2H), 2.32 (t, J = 7.4 Hz, 2H), 1.88 (quin, J = 7.1 Hz, 2H), 1.64 (dquin, J = 15.2, 7.4 Hz, 4H), 1.47 (quin, J = 7.6 Hz, 2H), 1.40 – 1.18 (m, 16H), 0.88 (t, J = 6.6 Hz, 3H)

Synthesis of undecyl 6-((2-hydroxyethyl)amino)hexanoate 15[7]

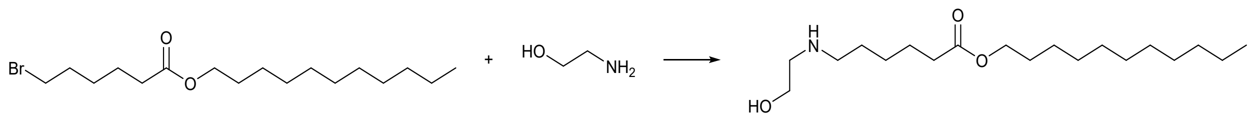

To an RBF is added undecyl 6-bromohexanoate **14** (15.03 g, 43.03 mmol), 2-MeTHF (80 ml), and DIPEA (6.1254 g, 47.23 mmol). This is stirred until a homogeneous solution is obtained before 4-Amino-1-butanol (20.3536 g, 333.23 mmol) and EtOH (80 ml) was added. The RBF is heated in a 60 °C oil bath for 16 hours. The RBF is allowed to cool to room temperature before the RBF is concentrated *in vacuo*. To the concentrated RBF is added EtOAc (100 mL) and HPW (100 mL). This is transferred to a separatory funnel where the contents are shaken and allowed to phase separate before separating the organic layer. The aqueous

is reextracted with EtOAc (50 mL) and all organic extracts are combined and dried (Na_2SO_4), and concentrated to give the salt as a off-white waxy solid (12.8917 g, 91%). A portion (9.00 g, 27.31 mmol) of this solid is purified via dissolution in EtOAc (50 mL), followed by addition of oxalic acid solution in EtOH (3.62 g, 28.68 mmol in 15 mL EtOH) giving a white precipitate at room temperature. A further portion of EtOAc (50mL) is added before the solid is filtered and washed with EtOAc (50 mL). This solid is transferred into a drying dish and is dried via vacuum oven (25 °C) with a slight nitrogen bleed to give the oxalate salt as a white fluffy powder (9.14 g, 80%). ^{1}H NMR (DMSO-d_6) is used to confirm structure before continuing. A portion of the white powder (6.0044 g, 14.31 mmol) is dissolved in 2-MeTHF (120 mL) and to this is added sat. $NaHCO_3$ (240 mL). This biphasic mixture is stirred at 50 °C for 1 hour before allowing to cool and transferring to a separatory funnel. The organic layer is separated before the aqueous is reextracted with 2-MeTHF (60 mL), the organic layers are combined, dried (Na_2SO_4), and concentrated to give the title compound as a white solid (4.53 g, 96%) (70% over the step).

Oxalate salt **17**:

^{1}H NMR (400 MHz, DMSO-d_6) δ 3.98 (t, J = 6.6 Hz, 2H), 3.63 (t, J = 5.3 Hz, 2H), 2.96 (t, J = 5.3 Hz, 2H), 2.90 – 2.82 (m, 2H), 2.28 (t, J = 7.4 Hz, 2H), 1.65 – 1.46 (m, 6H), 1.35 – 1.20 (m, 16H), 0.91 – 0.79 (m, 3H)

Title compound:

^{1}H NMR (400 MHz, $CDCl_3$) δ 4.06 (t, J = 6.6 Hz, 2H), 3.67 (t, J = 5.3 Hz, 2H), 2.81 (t, J = 5.1 Hz, 2H), 2.66 (t, J = 7.2 Hz, 1H), 2.32 (t, J = 7.4 Hz, 2H), 1.72 – 1.49 (m, 6H), 1.45 – 1.16 (m, 20H), 0.89 (t, J = 6.6 Hz, 3H)

Synthesis of heptadecan-9-yl 8-bromooctanoate 18[7]

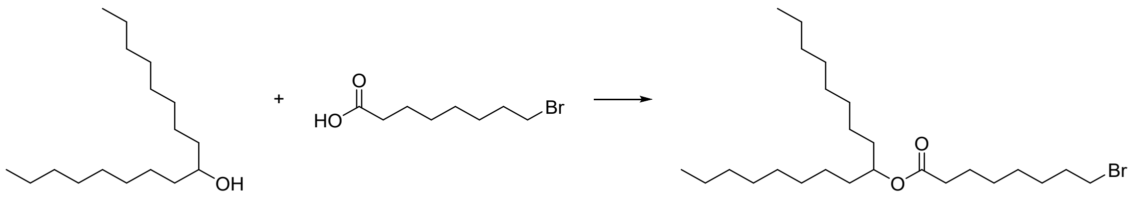

To an RBF is added 8-bromooctanoic acid (8.0929 g, 36.276 mmol), DMAP (219.4 mg, 1.793 mmol) followed by 2-MeTHF (60 mL). This is stirred until complete dissolution before cooling in an ice bath. To this is added NEt_3 (7.7624 g, 76.707 mmol), and PivCl (4.5416 g, 37.663 mmol) dropwise. This is stirred for 30 minutes before adding the alcohol as a mixture in 2-MeTHF (9.38 g, 36.57 mmol in 100 mL). The RBF is removed for the ice bath and allowed to stir and warm to room temperature. This is stirred for 23 hours before the contents for the RBF is emptied into a separatory funnel. The RBF is rinsed with sat. $NaHCO_3$ (50 mL) and added to the funnel before EtOAc (40 mL) is added to the funnel as well. This is shaken and organic layer is separated before drying (Na_2SO_4) and concentrating to give the title compound as a clear colorless oil. (17.48 g, 106%)

^{1}H NMR (400 MHz, $CDCl_3$) δ 4.87 (quin, J = 6.3 Hz, 1H), 3.41 (t, J = 6.6 Hz, 2H), 2.29 (t, J = 7.4 Hz, 2H), 1.85 (quin, J = 7.1 Hz, 2H), 1.73 – 1.57 (m, 2H), 1.56 – 1.18 (m, 44H), 0.88 (t, J = 6.6 Hz, 7H)

Synthesis of sodium 8-(heptadecan-9-yloxy)-1-hydroxy-8-oxooctane-1-sulfonate 19

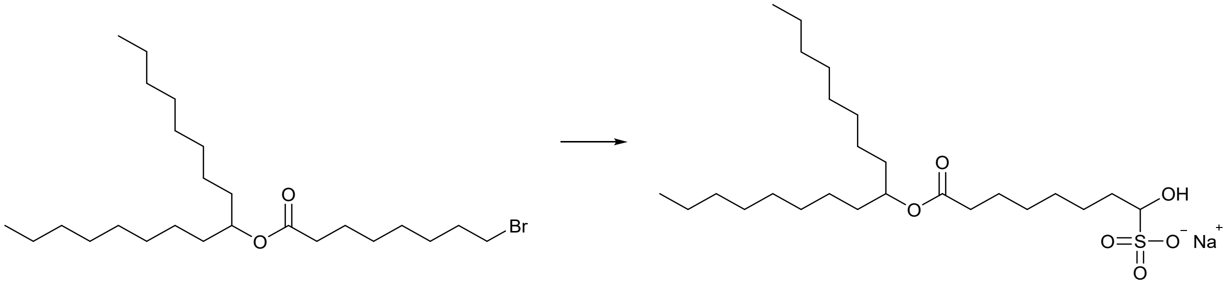

Compound was prepared using the General procedure for synthesis of fatty aldehyde bisulfite adducts. (5.100 g, 31%)

^{1}H NMR (400 MHz, DMSO-d_6) δ 4.77 (quin, J = 6.3 Hz, 1H), 3.76 (br dd, J = 9.4, 2.7 Hz, 1H), 2.24 (t, J = 7.2 Hz, 2H), 1.79 – 1.62 (m, 1H), 1.58 – 1.10 (m, 43H), 0.84 (br t, J = 6.6 Hz, 7H)

^{13}C NMR (101 MHz, DMSO-d_6) δ 172.54, 82.85, 72.92, 72.92, 33.82, 33.49, 31.25, 31.18, 28.78, 28.72, 28.52, 25.46, 24.66, 22.02, 13.85

2.2. Optimization of Bromide Oxidation Reaction Conditions

The initial trials of the reaction were performed in MeCN at 60 to 70 °C with 2-picoline *N*-oxide as the oxidant and a range of bases to determine feasibility as reported in a recent synthesis by Merck.[8] Sodium acetate provided the most promising result; amine bases formed significant amounts of alkyl bromide salts. Additionally, bases with a conjugate acid pKa greater than protonated 2-picoline did not form the required picolinium salt to sequester the 2-picoline byproduct, thus allowing for an increased amount of 2-picoline alkyl bromide salt to form over the course of the reaction. Performing the reaction in EtOAc provided comparable chemical yield and reaction time as MeCN, an expensive and relatively toxic solvent.

With this in mind, higher boiling solvents were evaluated to enable a higher reaction temperature and improve the overall kinetics of the reaction and to improve the yield (Table S2). While complete conversion was obtained in DMC as well as DEC after 16 hours, the workup with DMC was problematic due to its water miscibility and, therefore, the need to add additional organic solvent to extract the aldehyde product during aqueous workup. In doing so, emulsions became an issue. DEC was avoided due to its relatively high boiling point. n-Propyl acetate was investigated as an ideal solvent and was selected due to its higher boiling point improving kinetics, water azeotrope enabling azeotropic drying, and good properties with aqueous workup conditions. With general conditions established for the alkyl bromide oxidation to an aldehyde, process optimization proceeded in two improvement streams. One stream set out to intensify the general conditions chosen via human-in-the-loop statistical optimization, the other set out to probe slightly modified conditions which varied too much to include directly in the statistical optimization.

Statistically guided optimization via Bayesian optimization used a Gaussian surrogate model and probed the chemical space with the expected improvement acquisition function.[9] Inputs to the model included two categorical variables (*N*-oxide, and base) along with two continuous variables (*N*-oxide eq., and base eq.) and the result (yield) is optimized to find the maximum (Table S3). This led to an increase in the equivalents of *N*-oxide, which favored formation of the *N*-oxide adduct intermediate to

form the aldehyde product over the pyridinium alkyl bromide salt byproduct that sequesters the bromide starting material, therefore lowering the yield of aldehyde formation. The choice of oxidant was also evaluated. While the less hindered pyridine *N*-oxide provided increased reactivity compared to the 2-picoline *N*-oxide, it also had the downside of increased formation of undesired pyridinium alkyl bromide salt which leads to a slightly decreased yield (Table 3, iteration 3 entry 7, iteration 2 entry 8) with the caveat of reduced reaction times. Trimethyl amine *N*-oxide ($ONMe_3$), while productive in the reaction, did not give improvements over aromatic *N*-oxides. Pyridine *N*-oxide was chosen as the oxidant of choice as the amount of impurity did not affect any down-process steps, nor significantly impact yield. The large increase in *N*-oxide equivalents also led us to scale the reaction by a slow addition of bromide to the reaction mixture as a way to increase the effective concentration of *N*-oxide in the reaction relative to bromide **2** concentration, which would decrease the generation of the undesired pyridinium alkyl bromide salt. Addition of the bromide to the refluxing *N*-oxide, sodium acetate, n-propyl acetate solution over 4 or 8 hours gave similar increases to yield (^{1}H NMR) and decreases to the pyridinium impurity. As a result, 4 hours was chosen as the ideal dosing conditions. With these optimizations in place, an ^{1}H NMR yield of 88% was achievable with the main impurity being the pyridinium alkyl bromide salt.

Along with attempting traditional Ganem oxidation conditions,[10–12] slightly modified Kornblum conditions[13–16] were also evaluated but gave only 52% conversion and 30% yield (both via ^{1}H NMR) respectively after stirring for 72 h. Other *N*-oxides were also trialed (4-methylmorpholine *N*-oxide) but generated a significant amount of quaternary ammonium alkyl bromide salt regardless of the conditions. Finklestein-like conditions[17] were also investigated to increase the reaction rate and conversion but was found to only give 16% conversion after 27 h of reflux. Unfortunately, none of the probed conditions gave equivalent yields or conversions to the human-in-the-loop driven statistical optimization conditions which were used in all future bromide oxidation reactions

Table S2 Initial Bromide Oxidation Condition Screen

Entry	Temp.	Solvent[a]	*N*-oxide	Base	*N*-oxide eq.	Base eq.	Yield[b]
1	70	EtOAc	4-methylmorpholine	Na_2CO_3	4	5	52

2	60	DEC	pyridine	DIPEA	3	5	7
3	60	EtOAc	4-methylmorpholine	K_3PO_4	4	6	10
4	70	DEC	2-picoline	NaOAc	3	4	14
5	70	EtOAc	2-picoline	K_2CO_3	5	4	26
6	70	DEC	2-picoline	Cy_2NH	1.5	2	3
7	70	DEC	pyridine	$NaCO_3$	6	2	32
8	60	EtOAc	pyridine	Na_2CO_3	2	3	12
9	60	EtOAc	pyridine	NaOAc	5	3	20
10	92	DMC	2-picoline	NaOAc	2	1.5	68
11	110	DMC	2-picoline	NaOAc	6	2	72

[a] All reaction ran at 10V. [b] Determined by ^{1}H NMR. All reactions were run until full conversion is noted by ^{1}H NMR.

Trialed Finklestein-like conditions:

To an RBF with a condenser is added the bromide **2** (1.0136 g, 2.41 mmol) followed by acetone (10 mL), sodium iodide (0.0359 g, 0.2853 mmol), 2-picoline *N*-oxide (0.4077 g, 3.74 mmol), and sodium acetate (0.3934 g, 4.80 mmol). This was brought to reflux in an oil bath and monitored by ^{1}H NMR.

Trialed traditional Ganem oxidation conditions:

To an RBF is added bromide **2** (1.0028 g, 2.39 mmol), DMSO (10 mL), 4-methylmorpholine *N*-oxide (0.8389 g, 7.16 mmol) and finally NEt_3 (0.4891 g, 4.83 mmol). This was stirred at RT with monitoring via ^{1}H NMR.

Trialed traditional Kornblum oxidation conditions:

To an RBF is added bromide **2** (1.0301 g, 2.46 mmol), DMSO (10 mL) and NEt_3 (0.4976, 4.92 mmol). This was stirred at RT with monitoring via ^{1}H NMR.

Table S3 Bayesian Optimization of Ganem-type Oxidation by Iteration

**Iteration 1**	***N*-oxide**	**Base**	***N*-oxide eq**	**Base eq**	**Yield**[a]
1	NMe_3	Na_2CO_3	6	6	32
2	pyridine	K_3PO_4	3	2	52
3	pyridine	K_2CO_3	2	5	57
4	2-picoline	NaOAc	4	4	72
5	2-picoline	$NaHCO_3$	6	3	74

6	2-picoline	Na_2CO_3	2	3	53
7	pyridine	$NaHCO_3$	4	5	67
8	NMe_3	DIPEA	5	1.5	67
**Iteration 2**	***N*-oxide**	**Base**	***N*-oxide eq**	**Base eq**	**Yield**[a]
1	2-picoline	K_3PO_4	10	5	11
2	2-picoline	$NaHCO_3$	2	1.5	31
3	2-picoline	DIPEA	2	1.5	[b]35
4	2-picoline	K_2CO_3	10	1.5	38
5	pyridine	Na_2CO_3	2	3	59
6	2-picoline	DIPEA	10	1.5	65
7	2-picoline	$NaHCO_3$	10	2	70
8	2-picoline	$NaHCO_3$	10	1.5	75
**Iteration 3**	***N*-oxide**	**Base**	***N*-oxide eq**	**Base eq**	**Yield**[a]
1	pyridine	DIPEA	8	5	44
2	NMe_3	NaOAc	2	1.5	49
3	NMe_3	NaOAc	9	4	[b]50
4	NMe_3	K_3PO_4	9	1.5	[b]52
5	pyridine	NaOAc	2	2	56
6	pyridine	$NaHCO_3$	2	6	56
7	pyridine	$NaHCO_3$	10	1.5	67
8	pyridine	NaOAc	10	1.5	90

[a] Determined by ^{1}H NMR. [b] Experimental issues experienced include reaction drying over length of reflux. All reactions were refluxed for 16 hours. All reactions ran at 10V. Range of continuous variables: *N*-oxide eq. = 2 to 10 increments of 1, Base eq. = 1.5, 2, 3, 4, 5, 6. Batch size = 8.

2.3. Aldehyde Purification Metrics

Table S4 Purification Process Comparison Between Previous Traditional Column Chromatography and Bisulfite Adduct Precipitation of Crude Aldehyde **3** From TEMPO Oxidation

Purification Process	Crude Aldehyde Input	Aldehyde Recovery	Purity (%)	PMI
**Column Chromatography**	94.46g	33.97g (36%)	99.1	373.0
**Bisulfite Adduct Precipitation**	53.09g	33.08g (62%)	83.0	27.2

Table S5 Performed Bisulfite Adduct Precipitation Scales

Bisulfite Adduct Intermediate	Anti-solvent used	Scale (Product)	Adduct Yield
**4 (From 9)**	DMC	42.79g	90%
**4 (From 2)**	Ethyl Formate	23.86g	73%
**13**	Ethyl Formate	9.67g	58%
**19**	Ethyl Formate	5.100g	31%

2.4. Formulation Results

Table S6 Physiochemical formulation results from previous formulation batches (PG893-177 and PG893-181) along with formulation results using ALC-0315 **1** from **9** (BSS-981-078)

Analysis	PG893-177 (TT batch#1)	PG893-181 (TT batch #2)	BSS-981-078
**Z-Avg (nm)**	71.17	71.36	73.28
**PDI**	0.08	0.08	0.07
**Encapsulation Efficiency**	96%	95%	97%

2.4. Bisulfite Adduct Stability Study

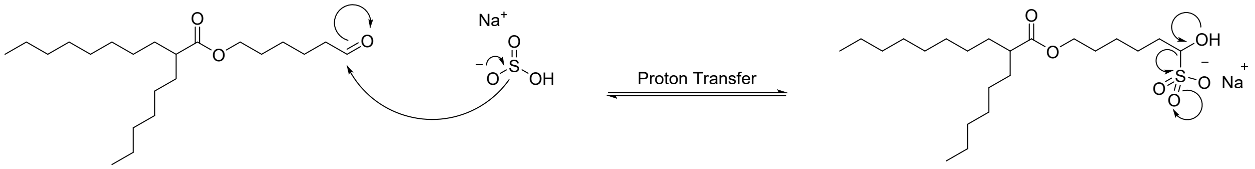

Scheme S3 Bisulfite Adduct Formation Mechanism

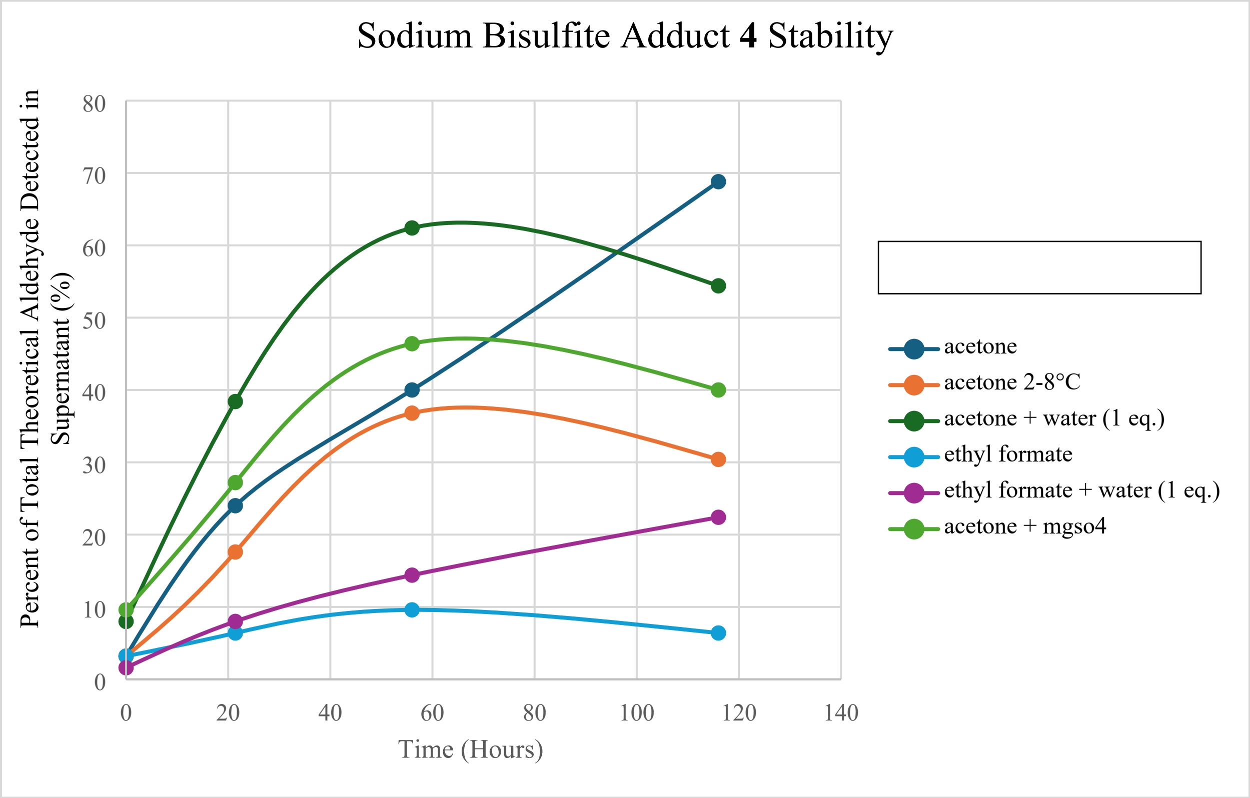

Figure S1 Bisulfite adduct **4** stability in various conditions and solvents.

3.1. ^{1}H NMR Spectra

ALC-0315 1 (From 6-hydroxyhexyl 2-hexyldecanoate **9**)[3]

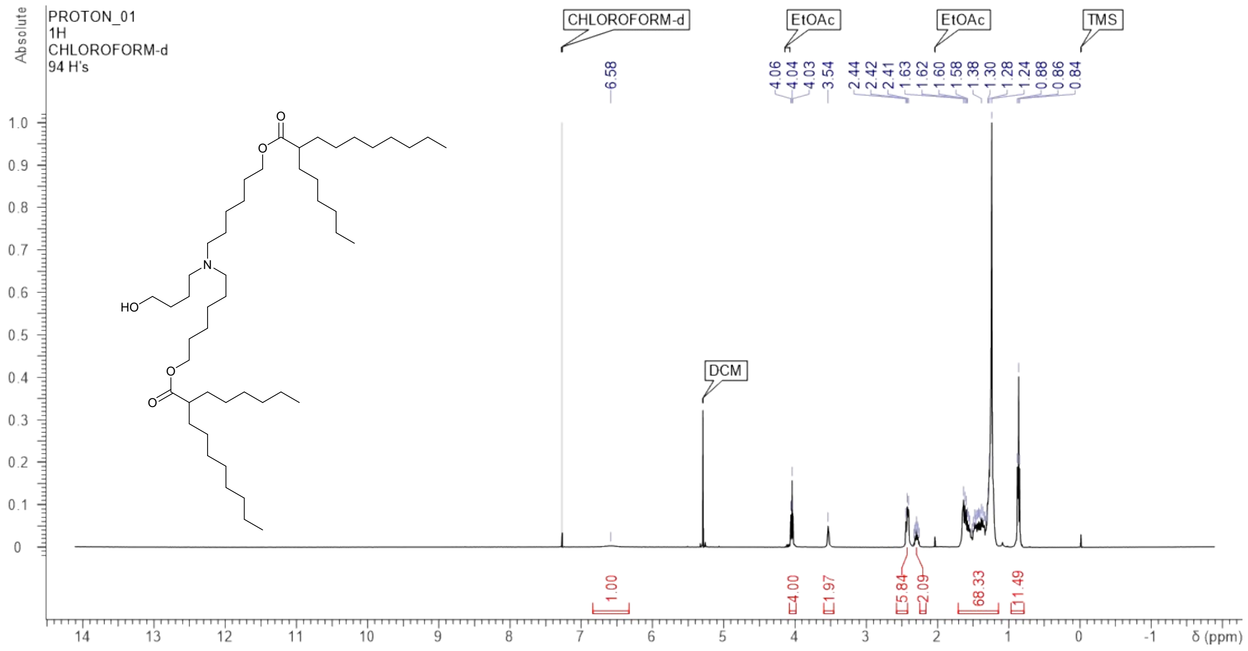

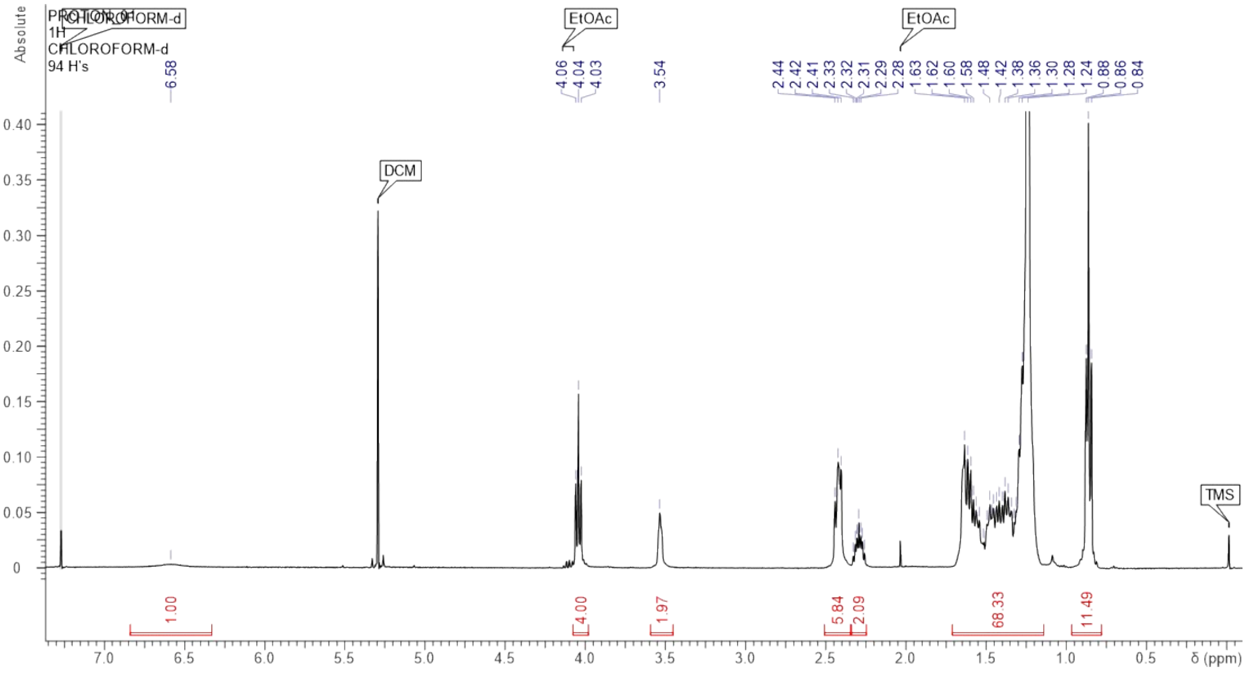

6-bromohexyl 2-hexyldecanoate 2[2]

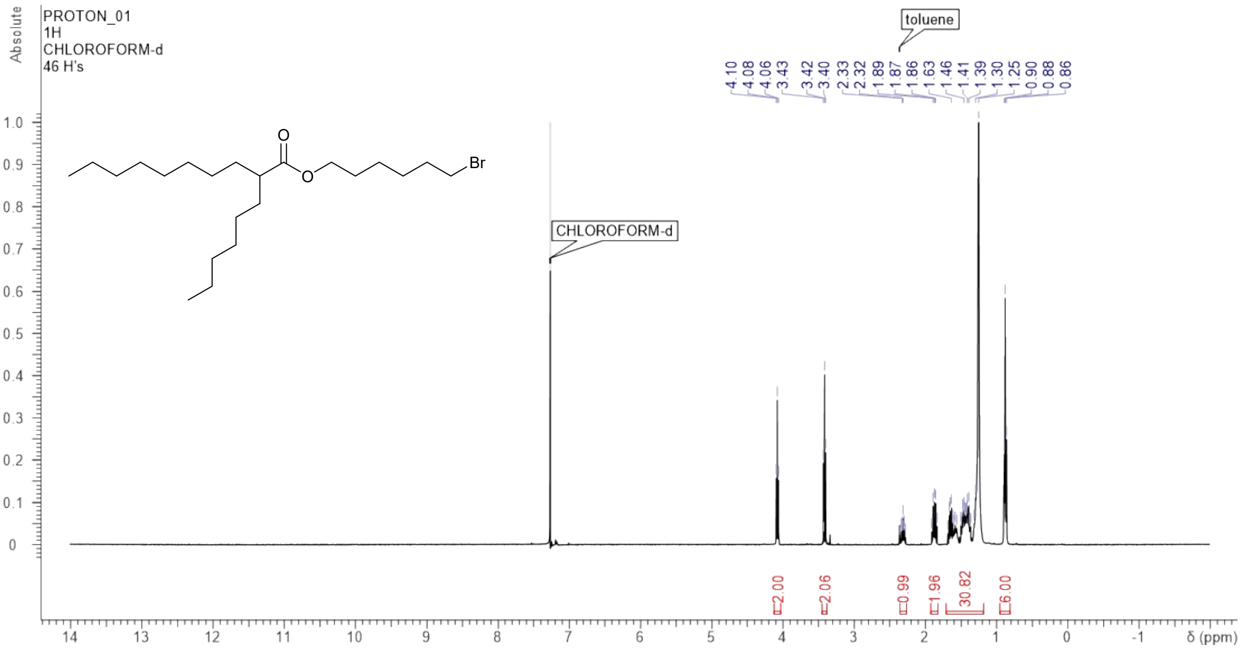

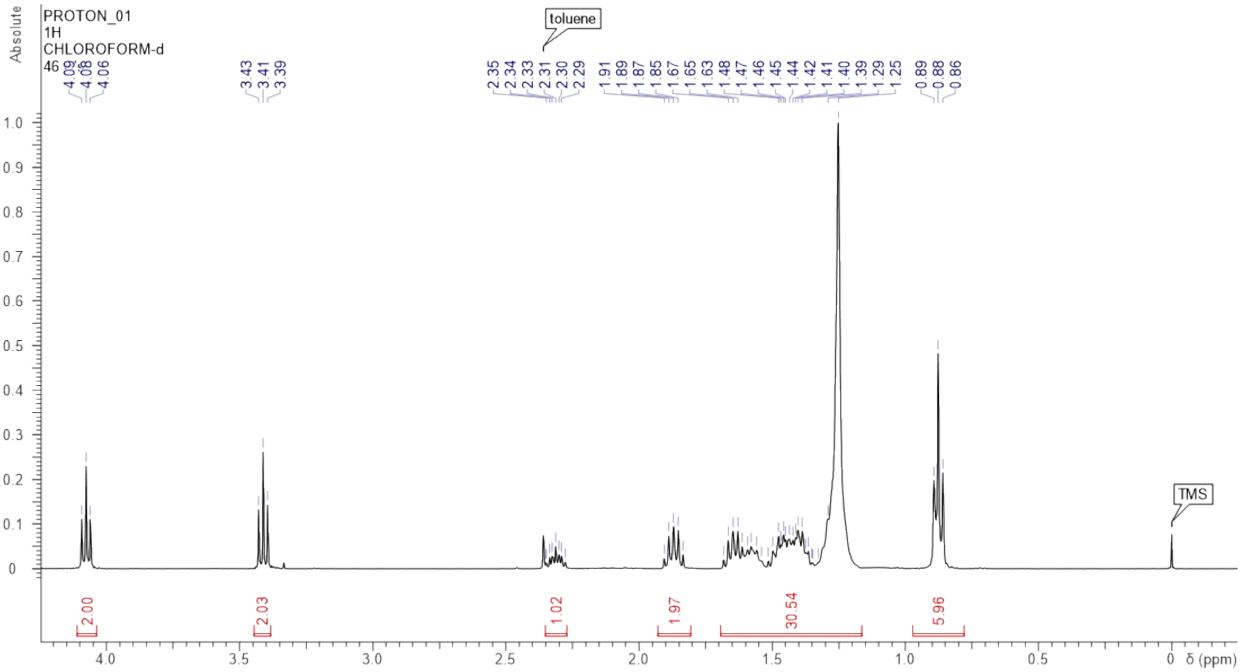

sodium 6-((2-hexyldecanoyl)oxy)-1-hydroxyhexane-1-sulfonate 4

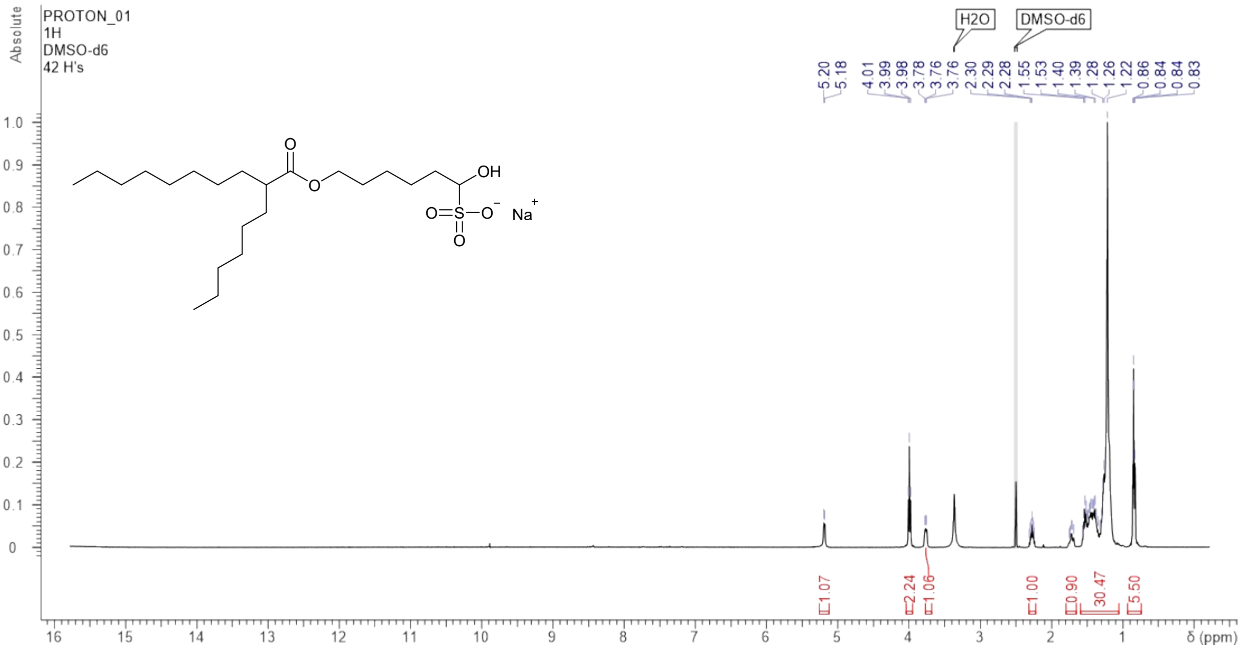

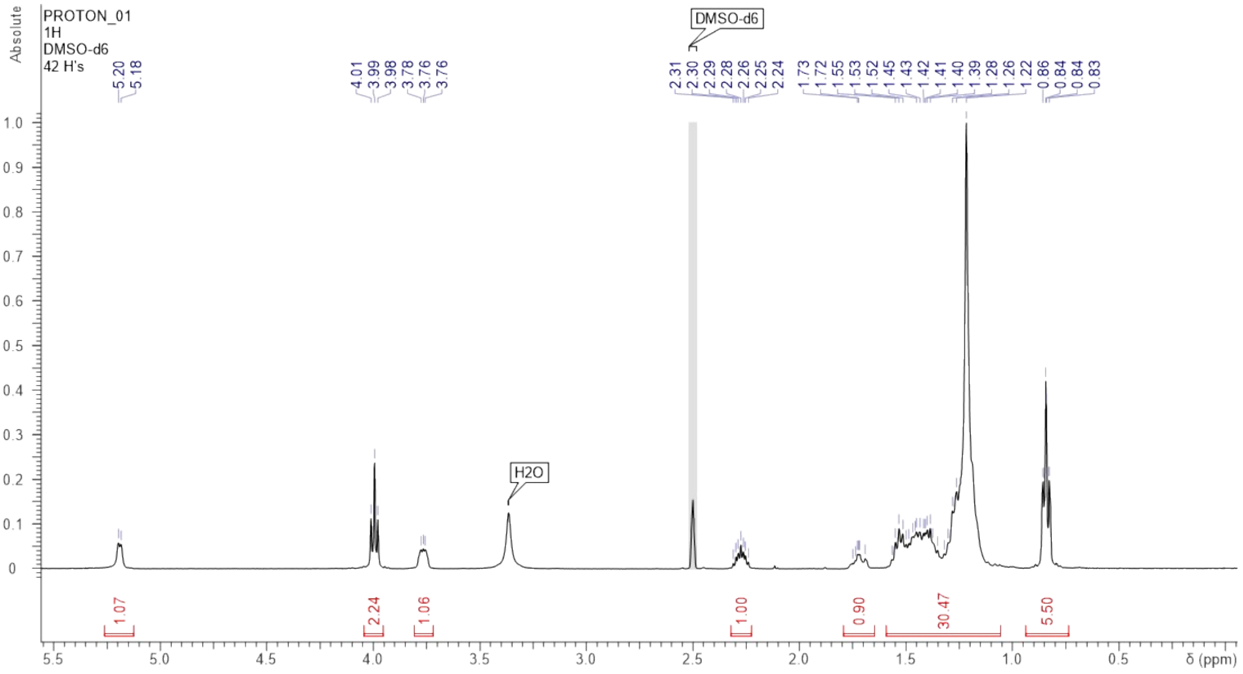

FTT5 5[4]

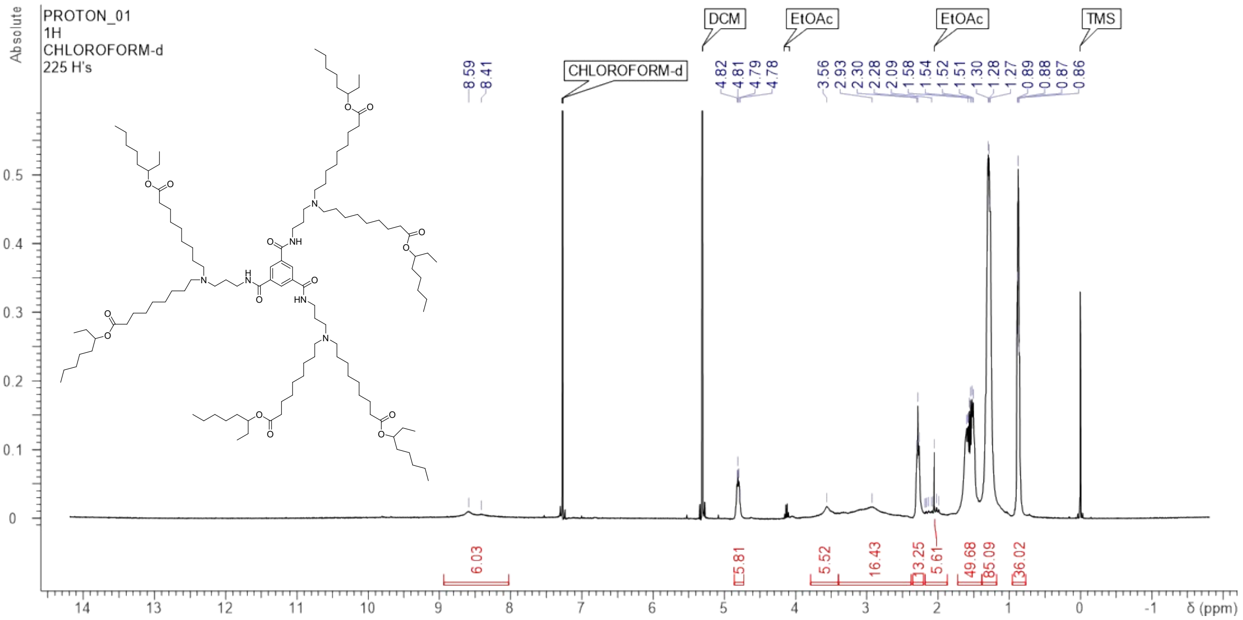

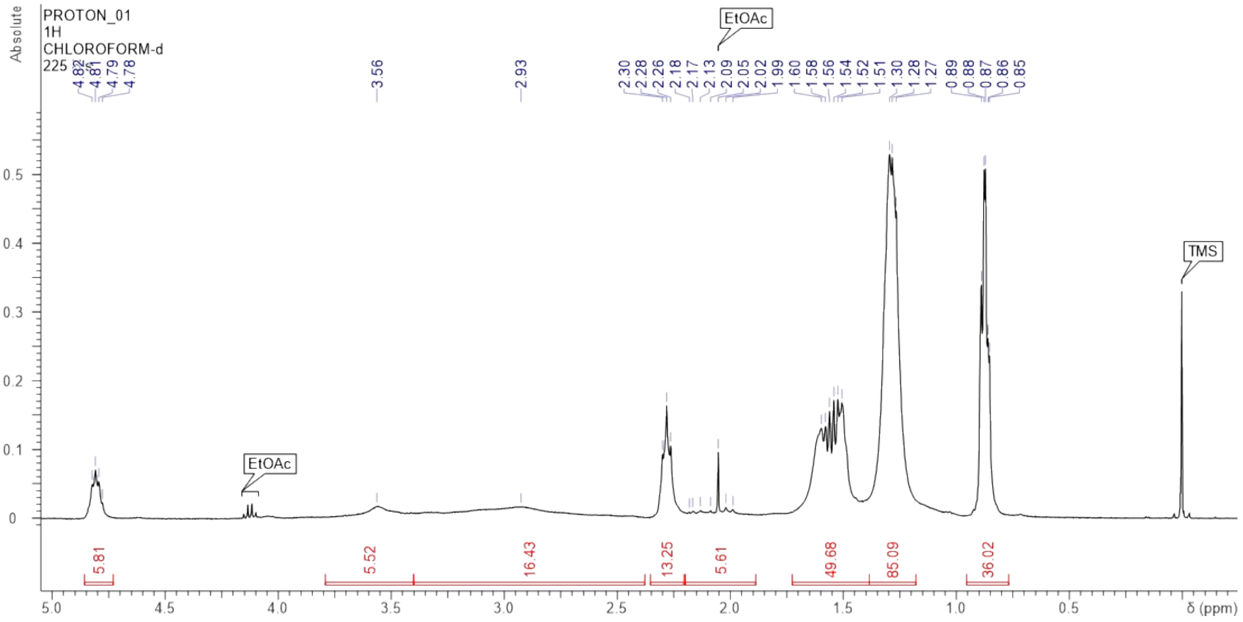

CH_2 signals adjacent to nitrogens and internal to the phenyl ring are notably broad.

SM-102 6[7]

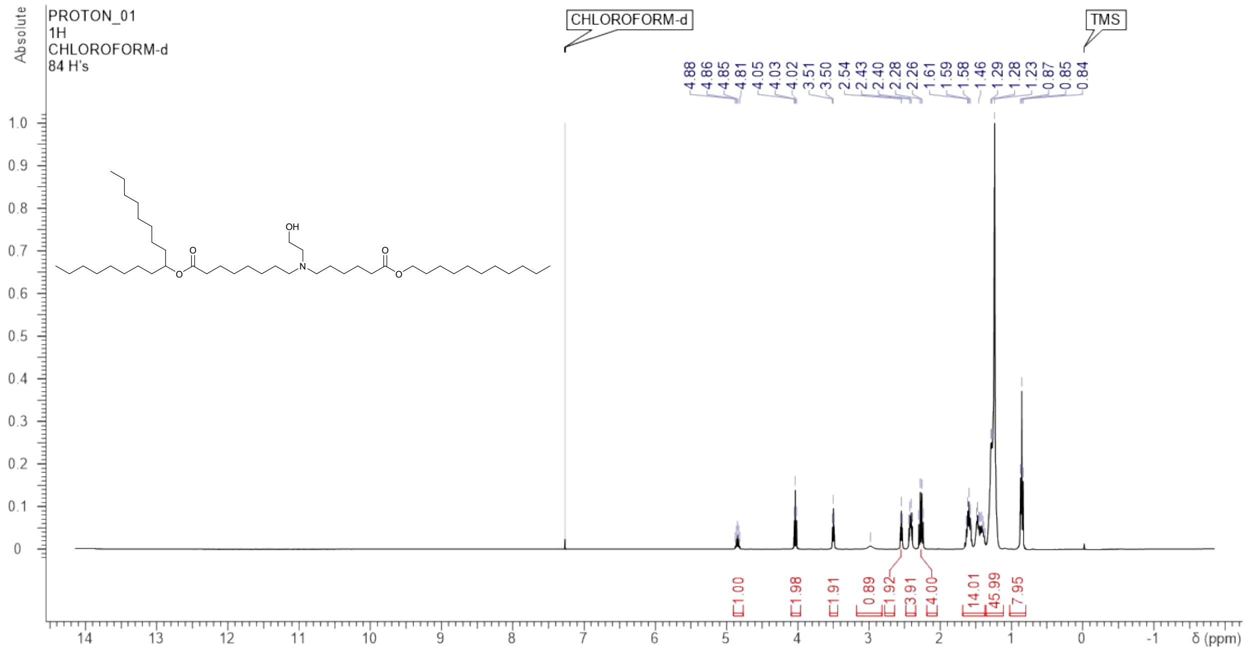
PROTON_01
1H
CHLOROFORM-d
84 H's
CHLOROFORM-d
TMS
Absolute
δ (ppm)

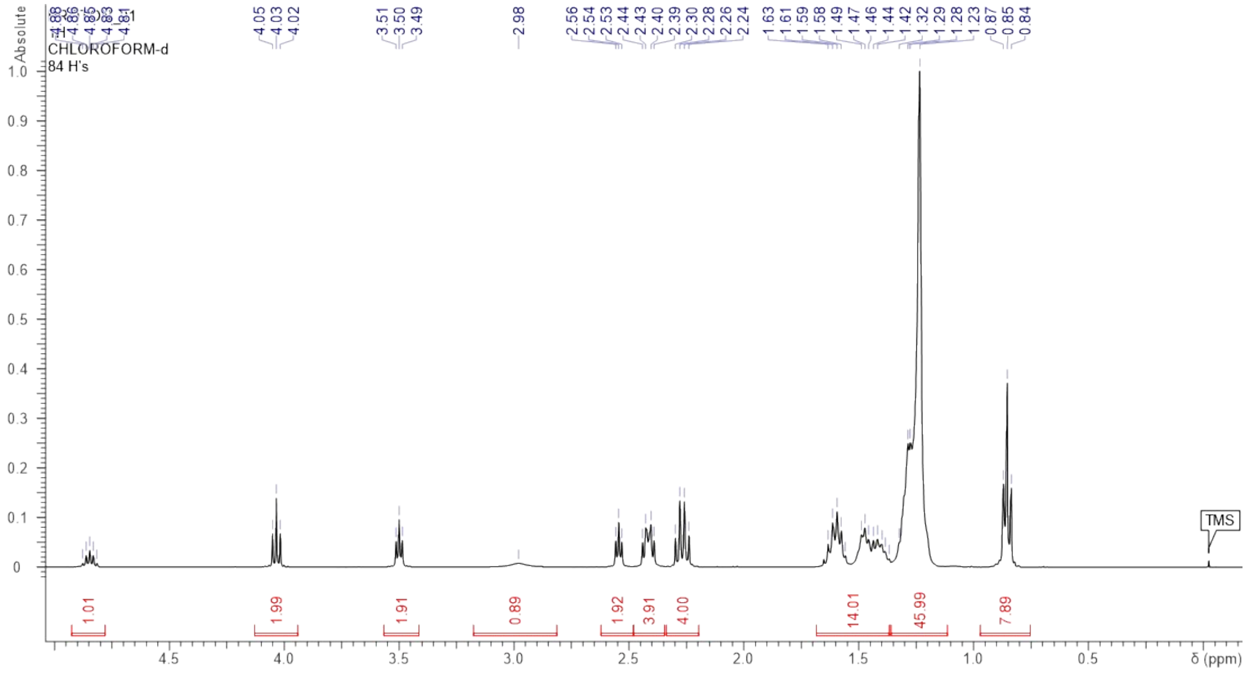
CHLOROFORM-d
84 H's
TMS
Absolute
δ (ppm)

6-hydroxyhexyl 2-hexyldecanoate 9[3]

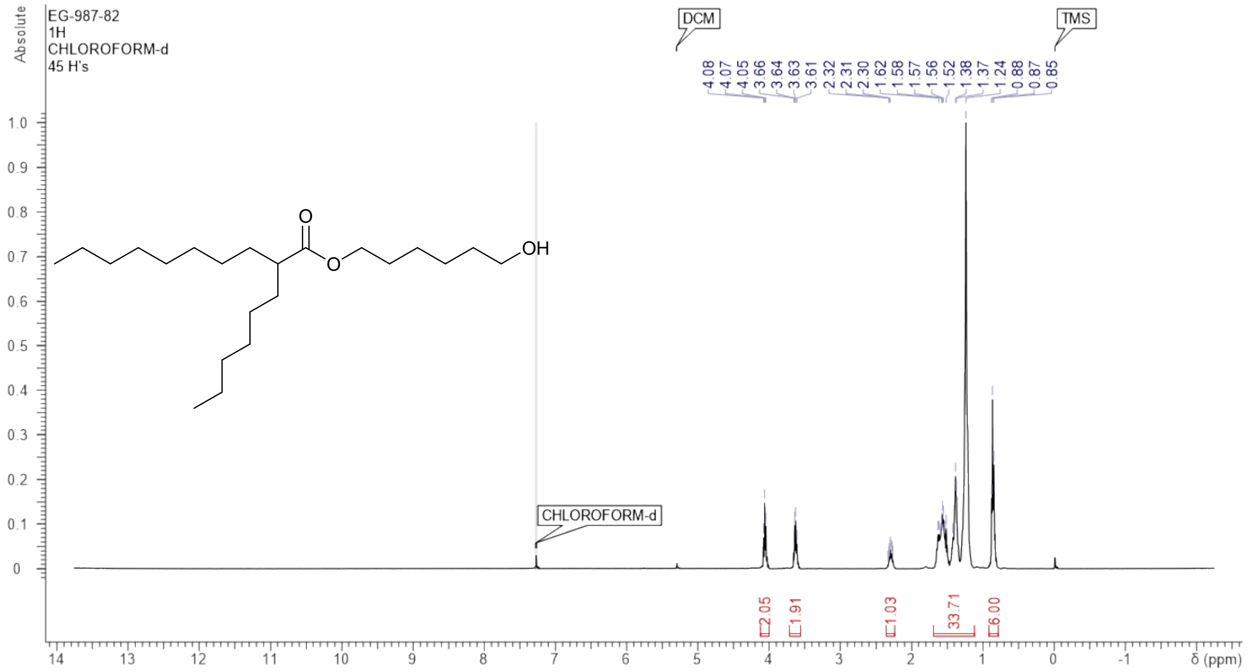

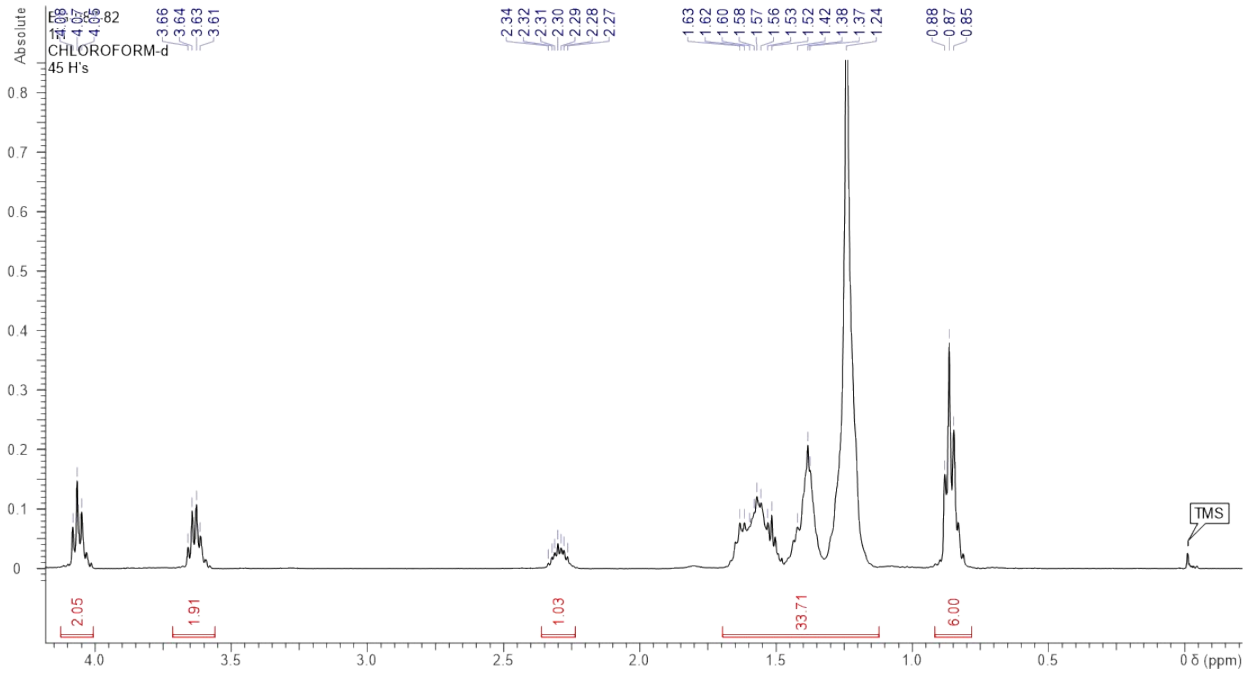

N1,N3,N5-tris(3-aminopropyl)benzene-1,3,5-tricarboxamide 10[5]

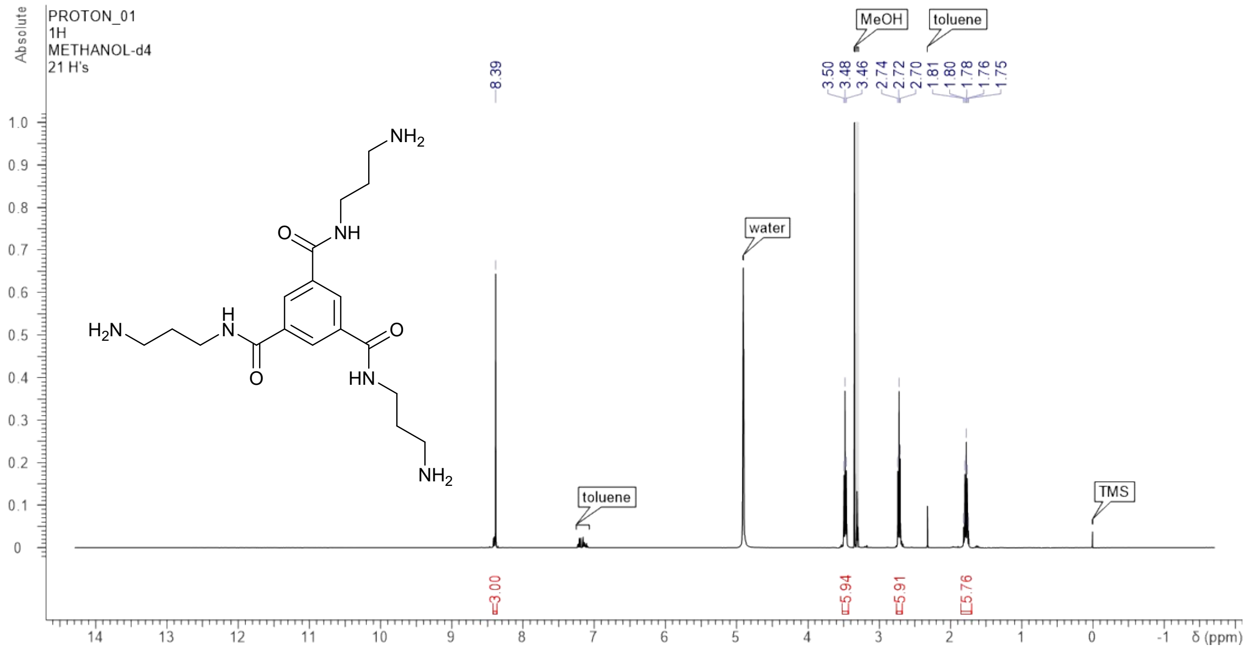

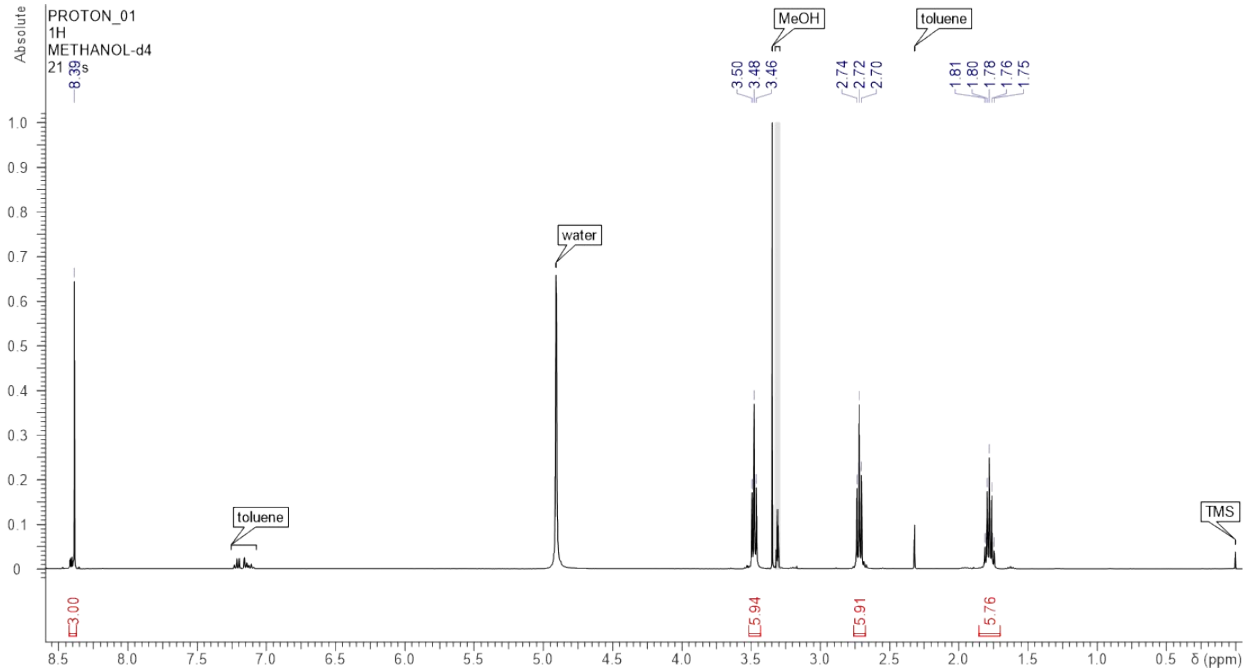

octan-3-yl 9-bromononanoate 12[6]

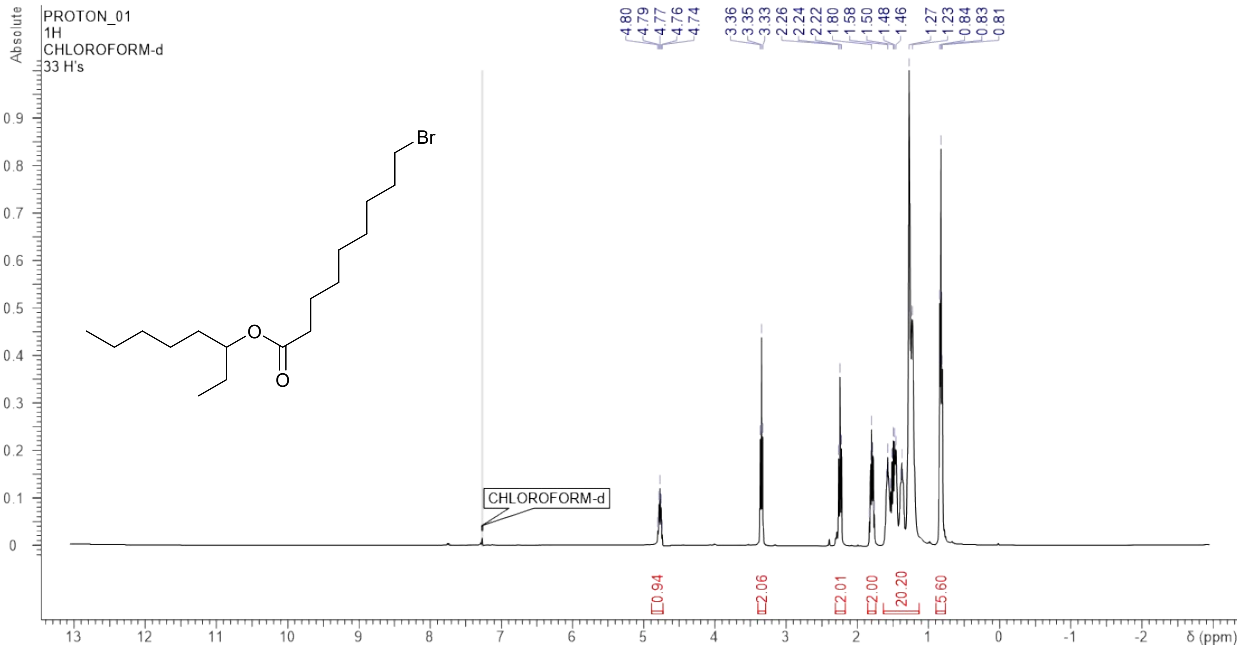

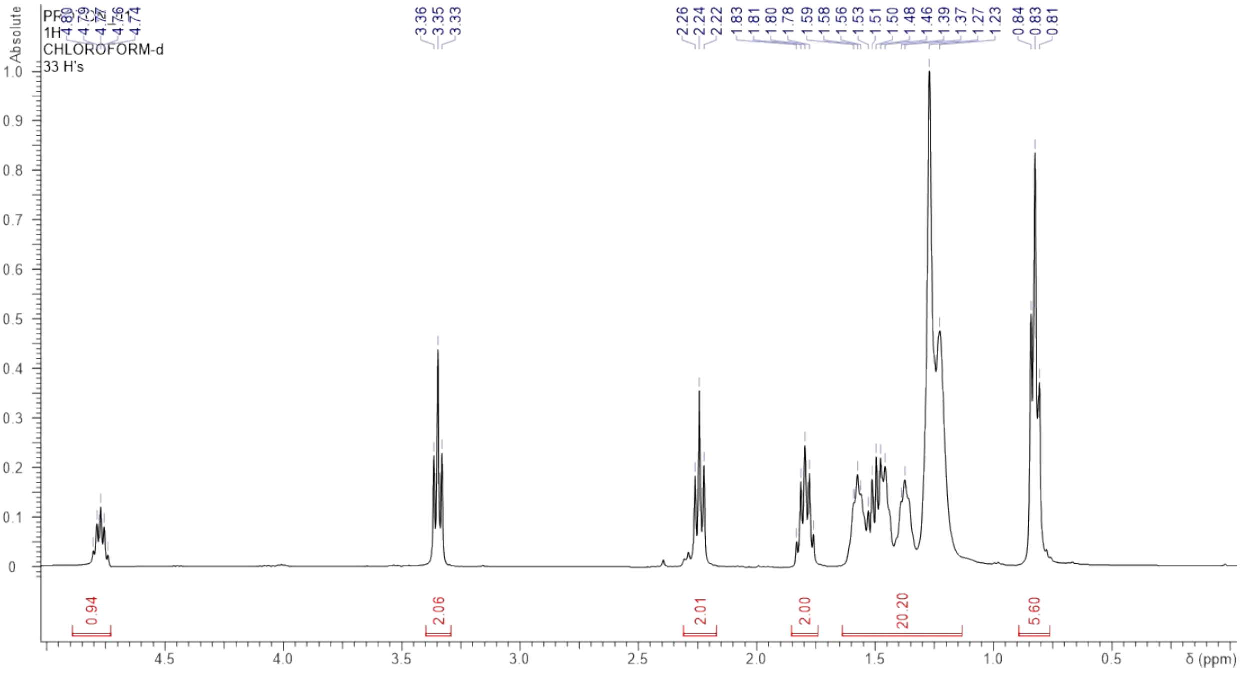

sodium 1-hydroxy-9-(octan-3-yloxy)-9-oxononane-1-sulfonate 13

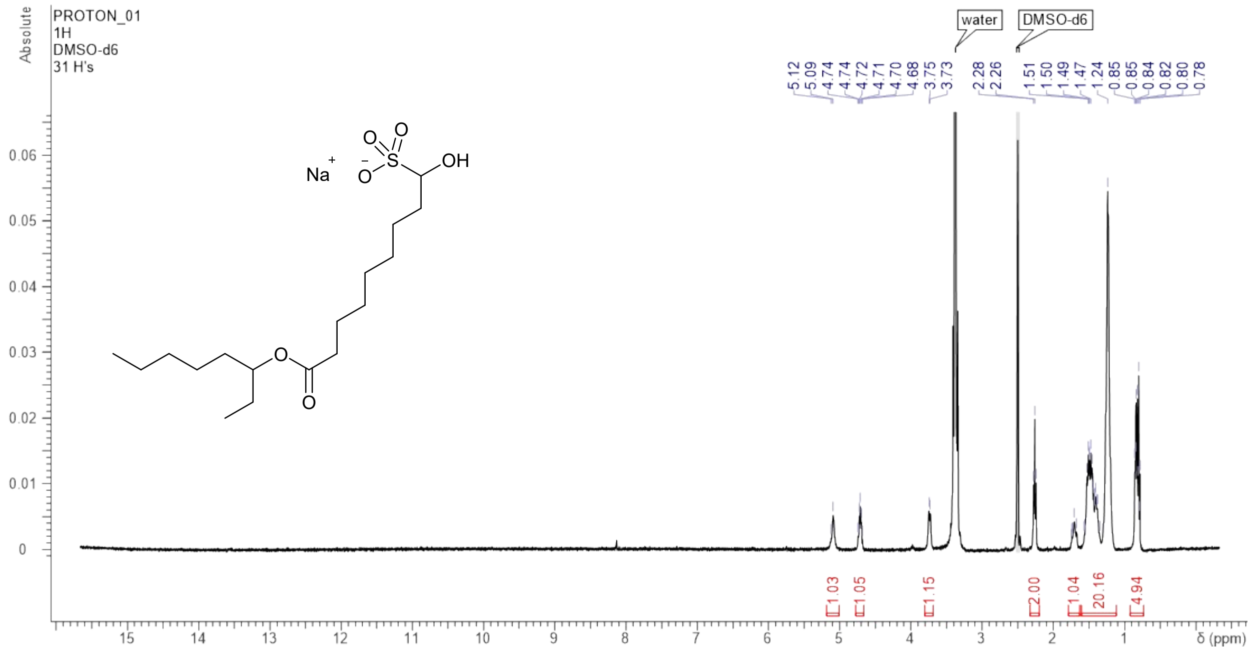

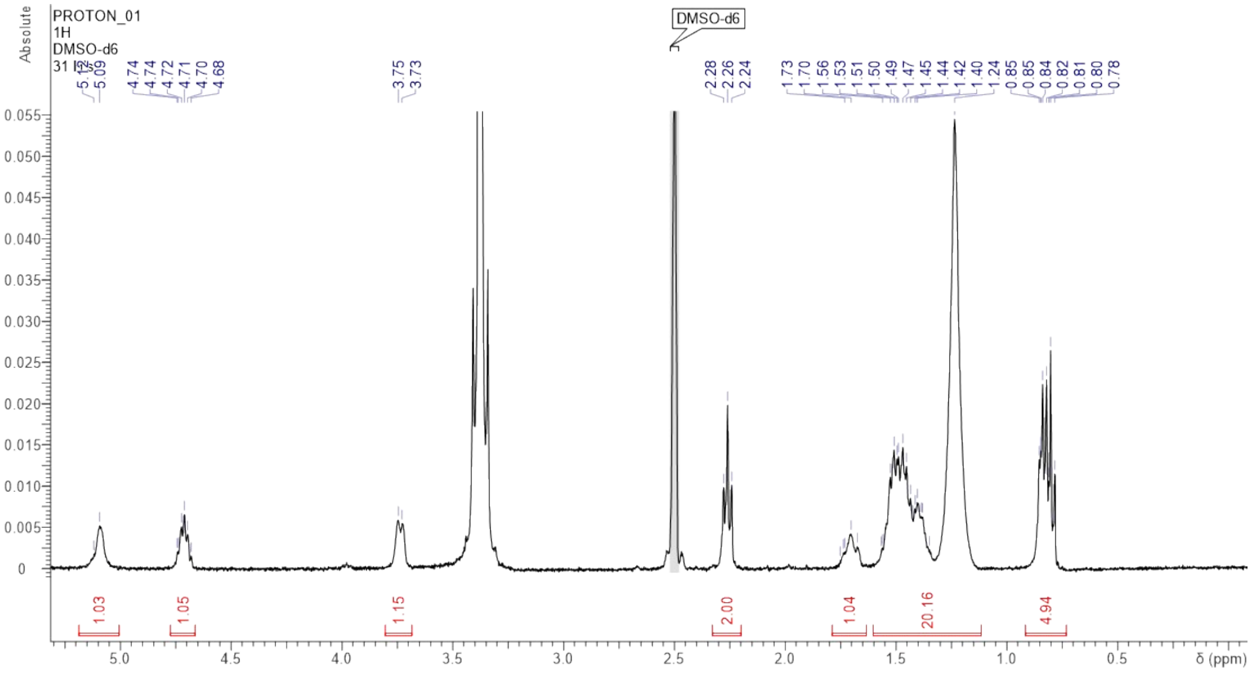

undecyl 6-bromohexanoate 14[7]

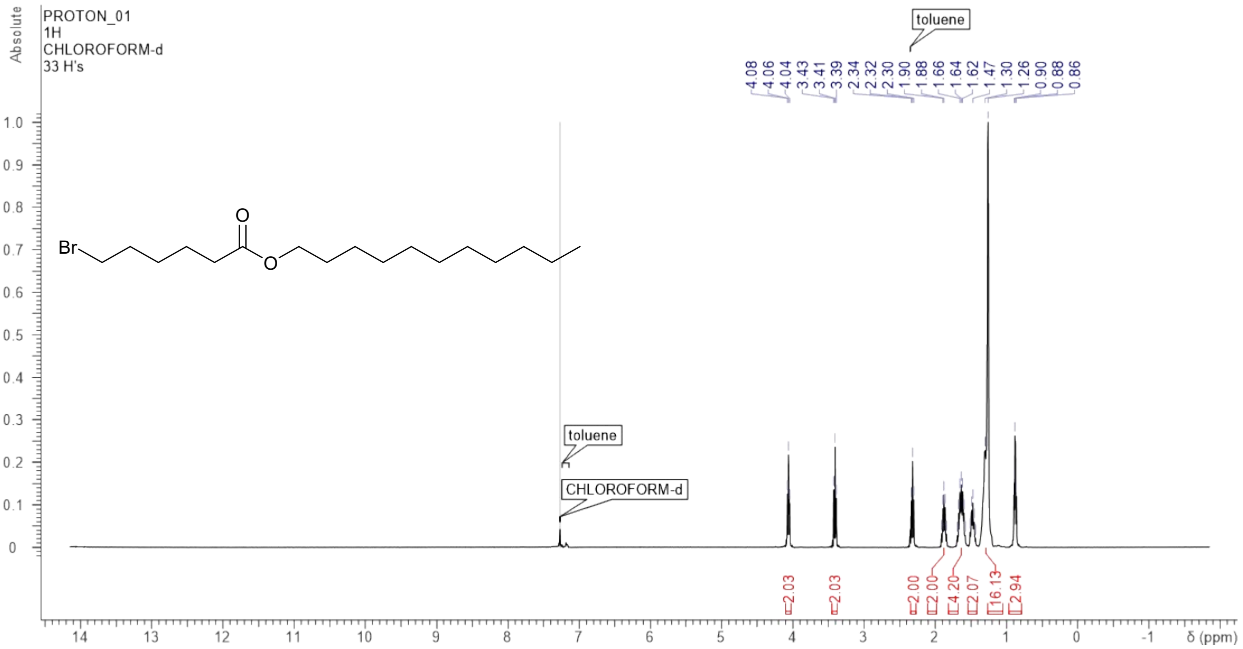

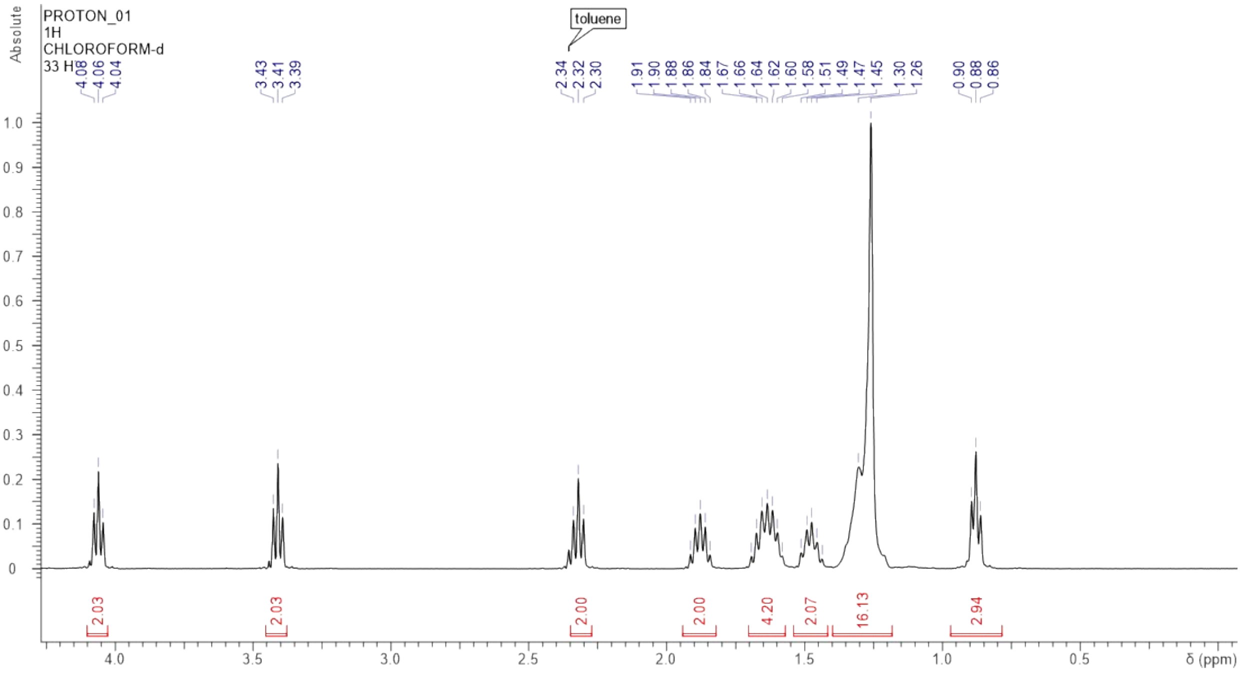

undecyl 6-((2-hydroxyethyl)amino)hexanoate 15[7]

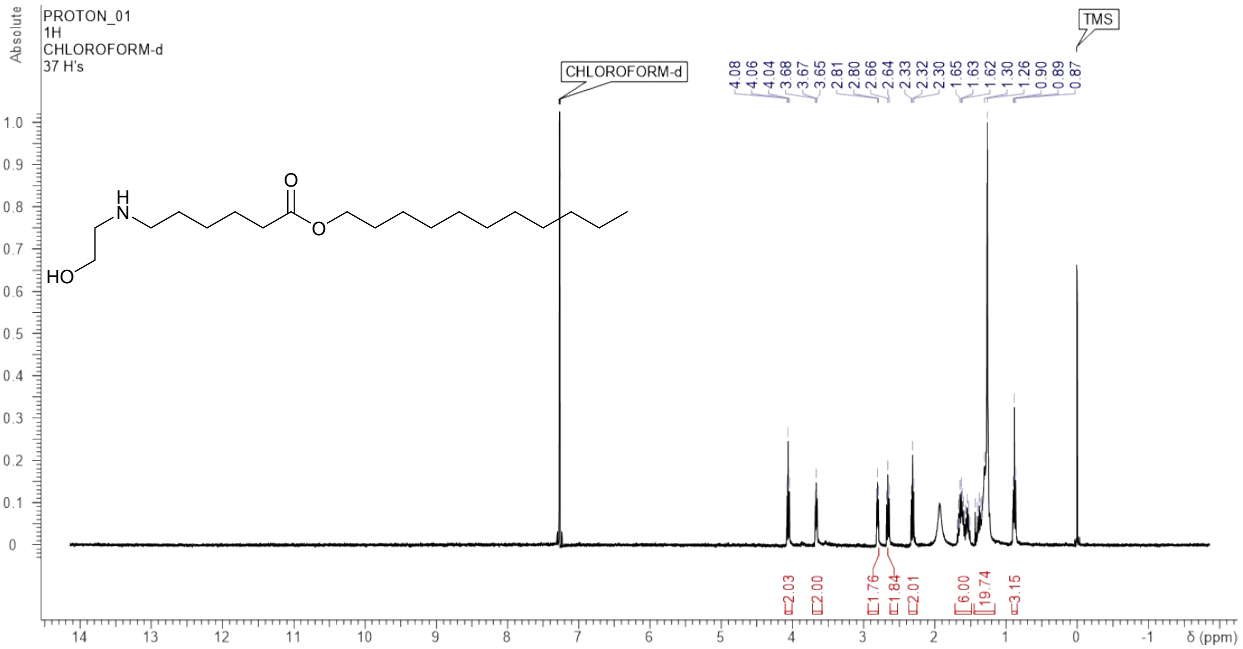

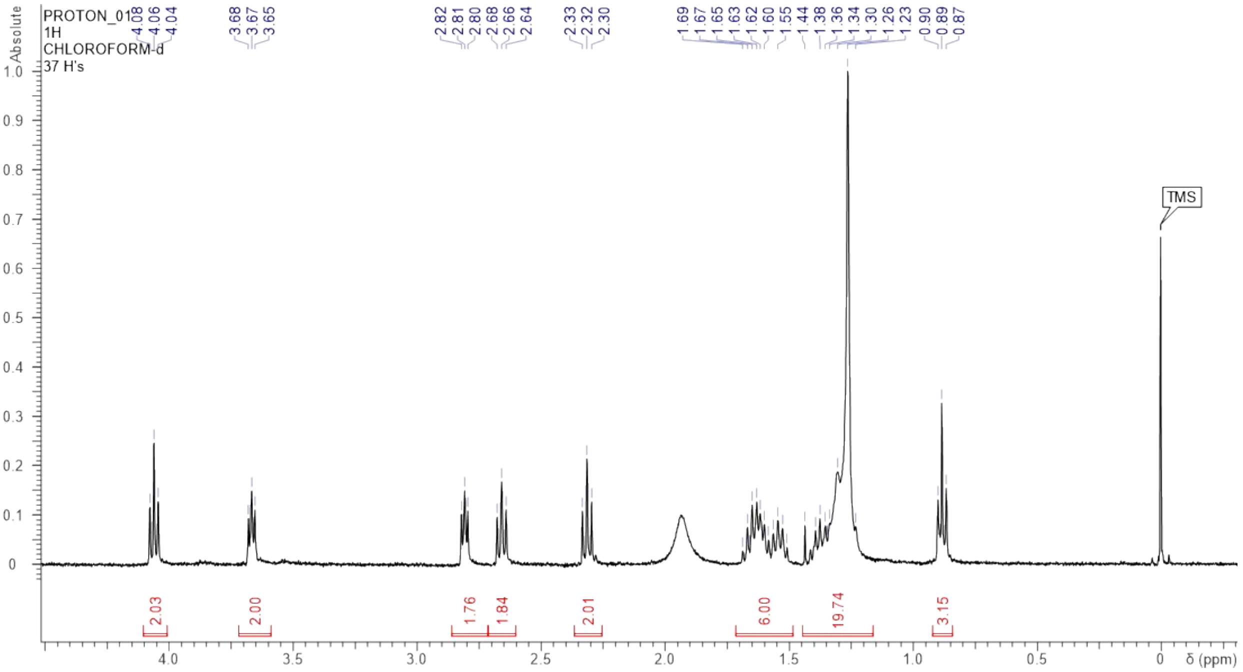

undecyl 6-((2-hydroxyethyl)amino)hexanoate oxalate 17

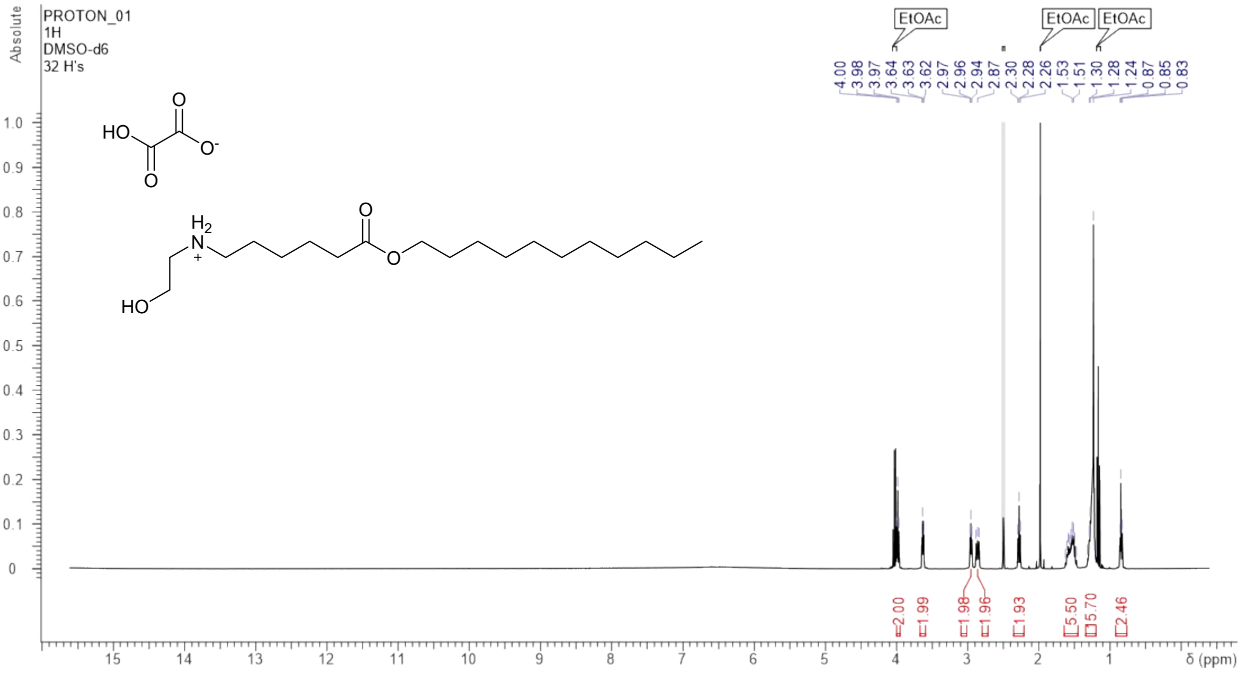

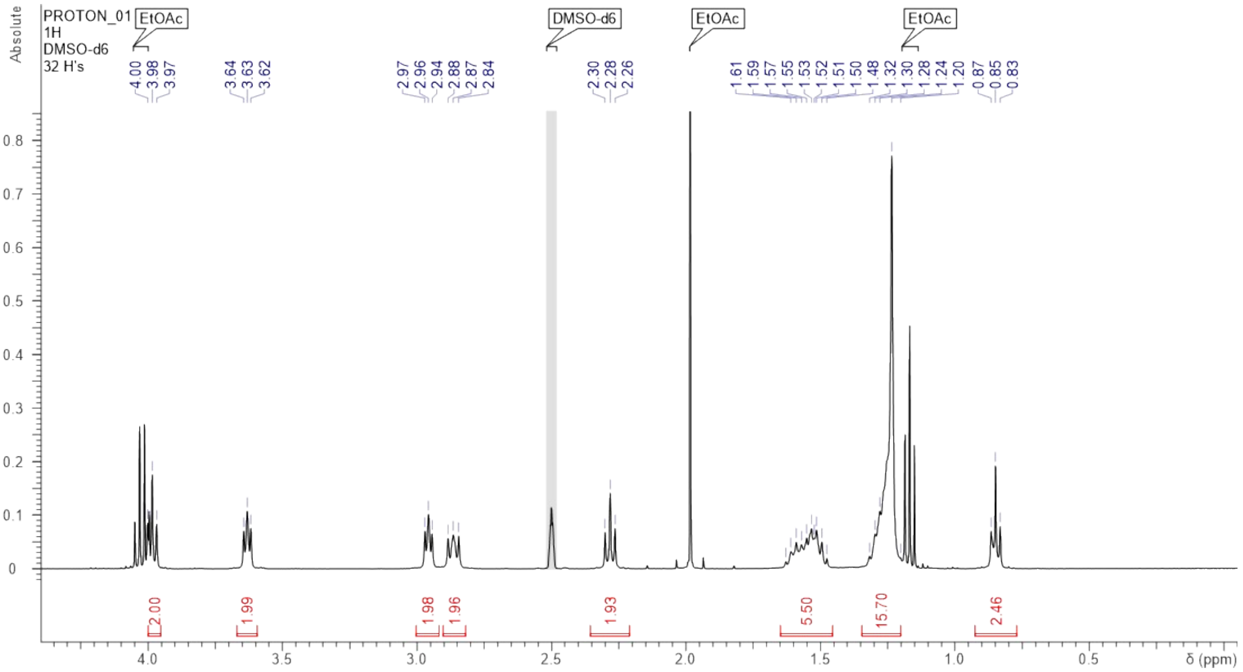

heptadecan-9-yl 8-bromooctanoate 18[7]

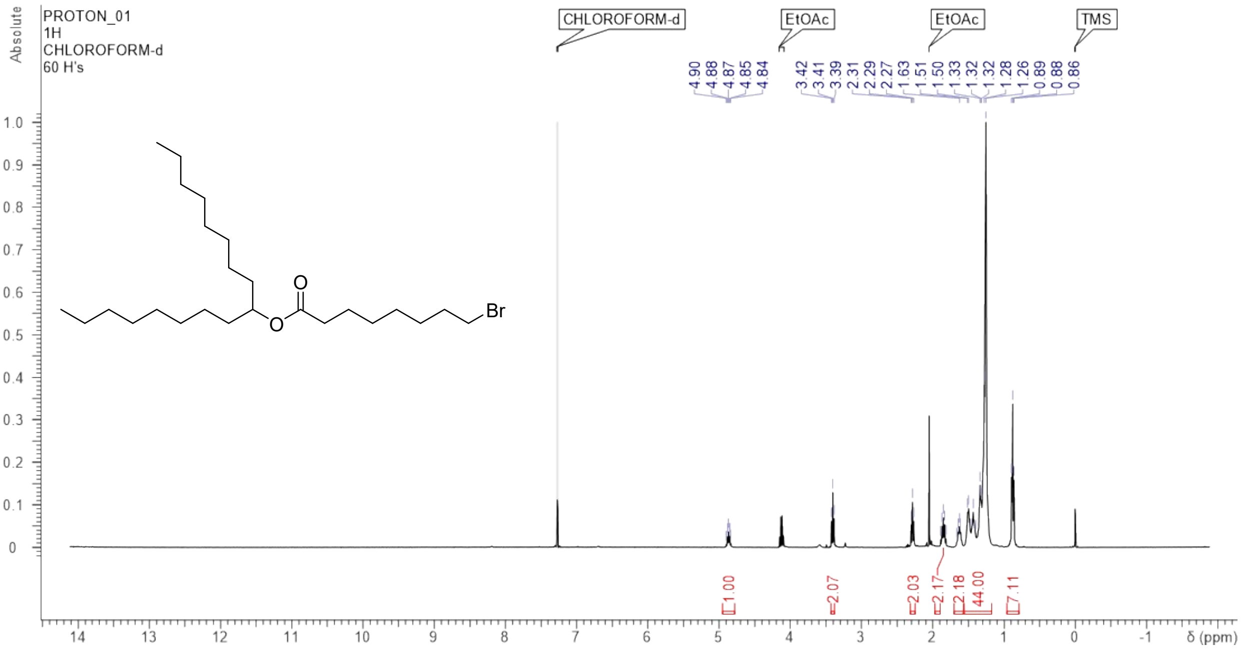

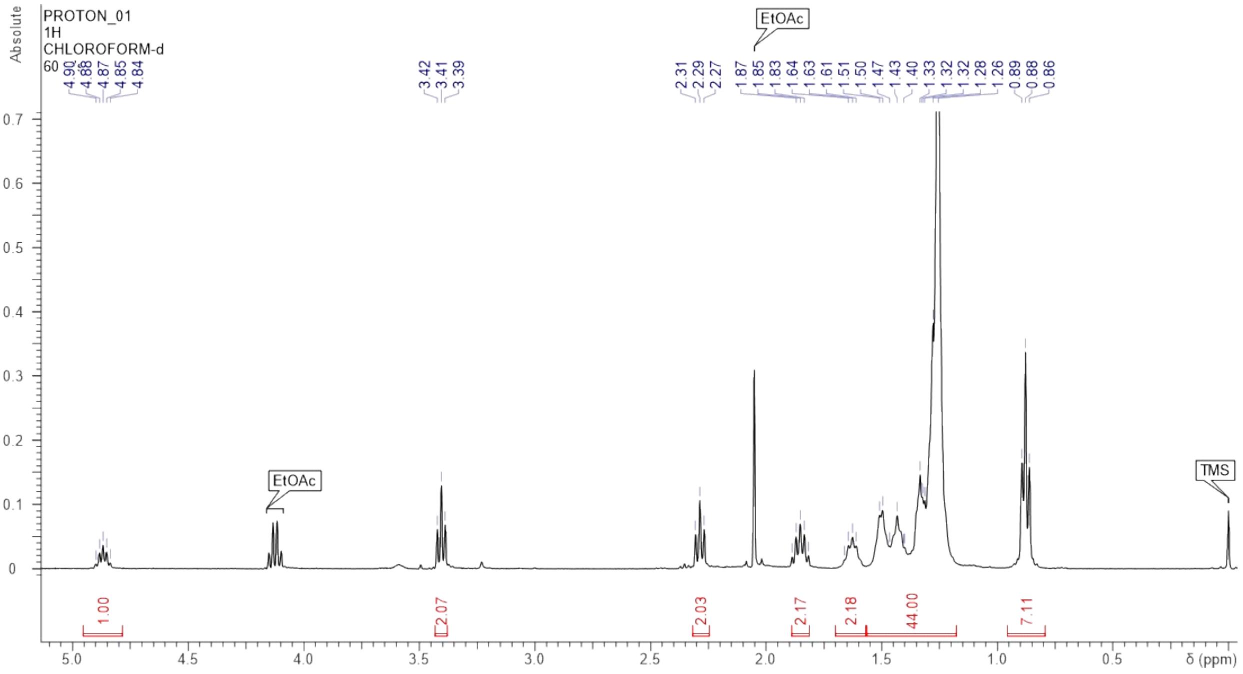

sodium 8-(heptadecan-9-yloxy)-1-hydroxy-8-oxooctane-1-sulfonate 19

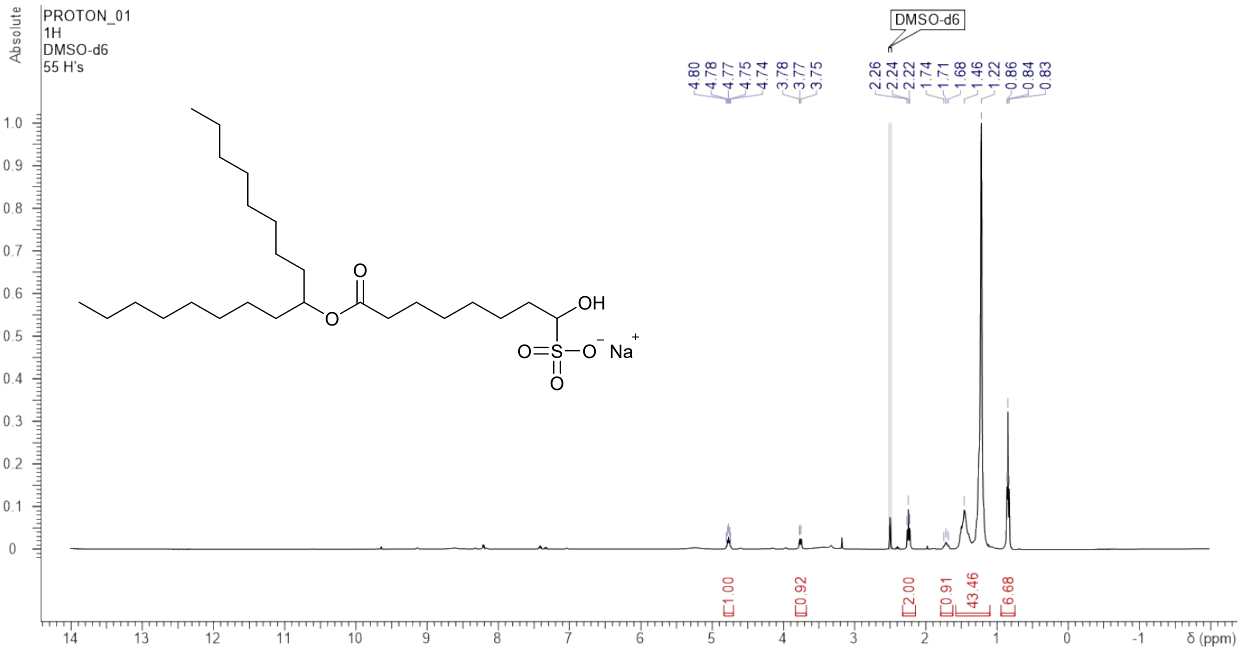

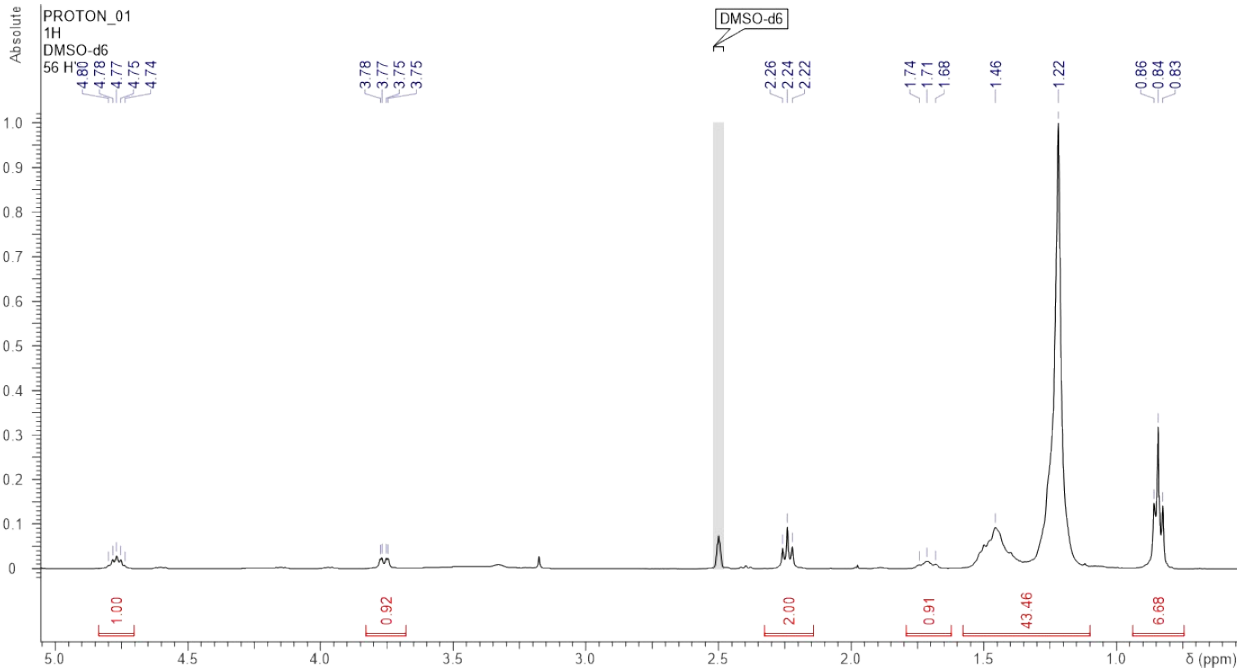

4.1. ^{13}C NMR Spectra

sodium 6-((2-hexyldecanoyl)oxy)-1-hydroxyhexane-1-sulfonate 4

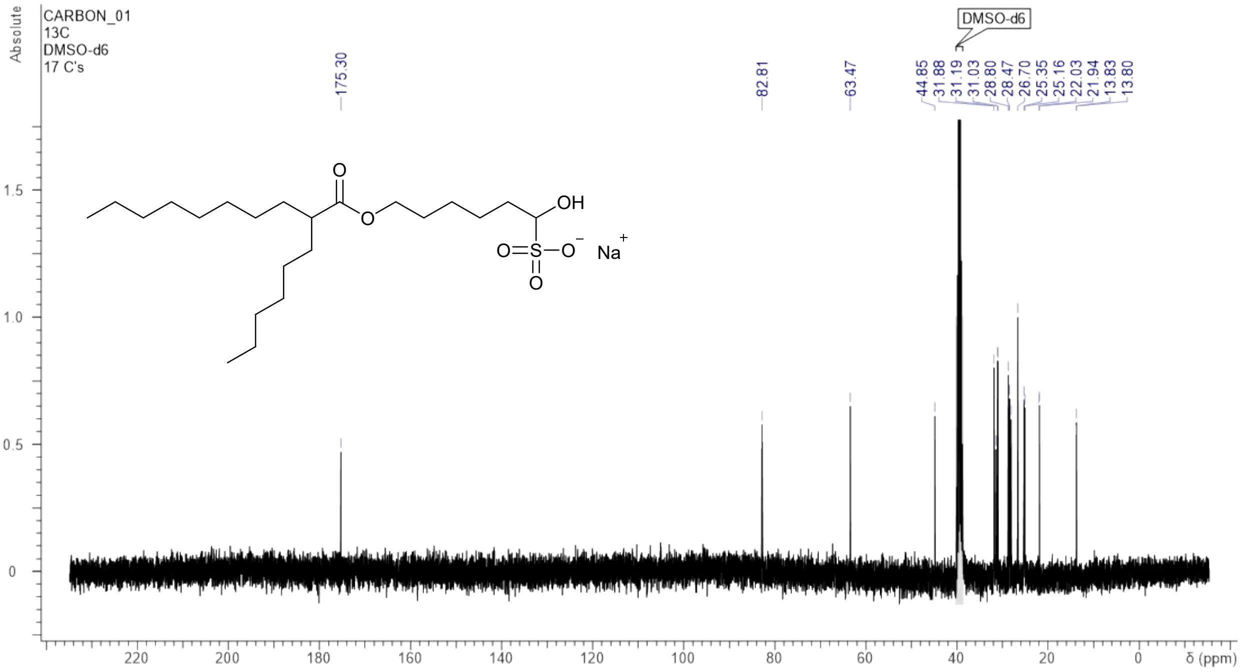

sodium 1-hydroxy-9-(octan-3-yloxy)-9-oxononane-1-sulfonate 13

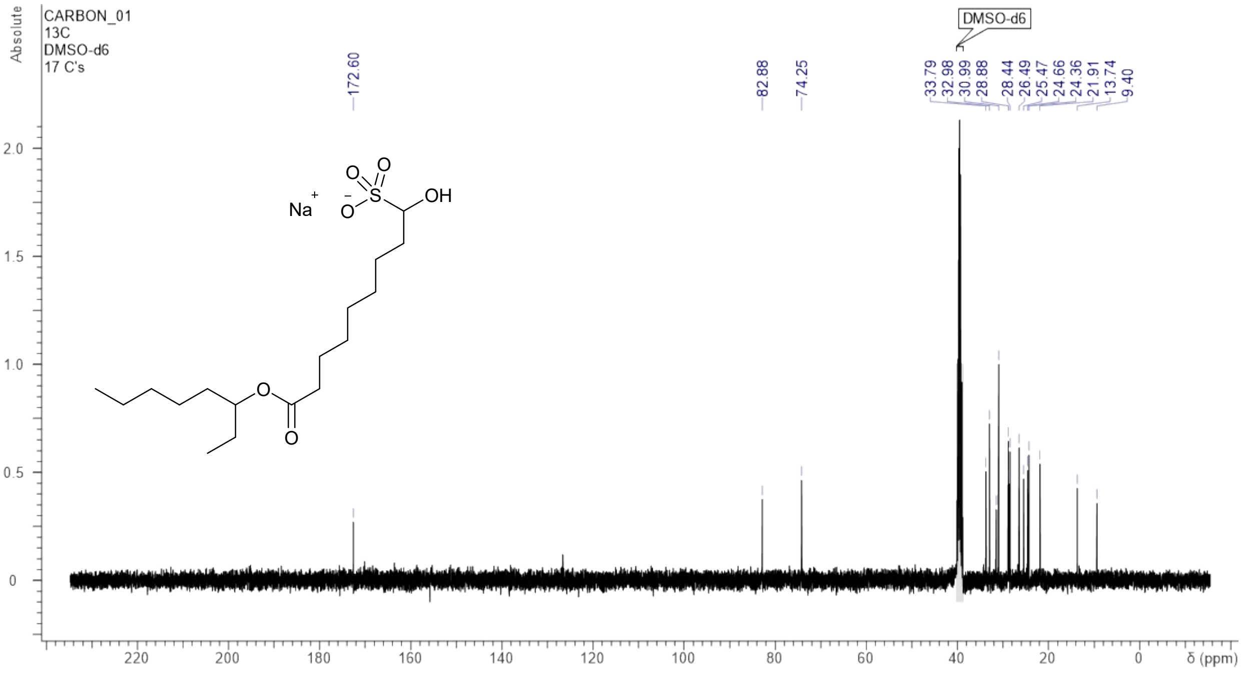

sodium 8-(heptadecan-9-yloxy)-1-hydroxy-8-oxooctane-1-sulfonate 19

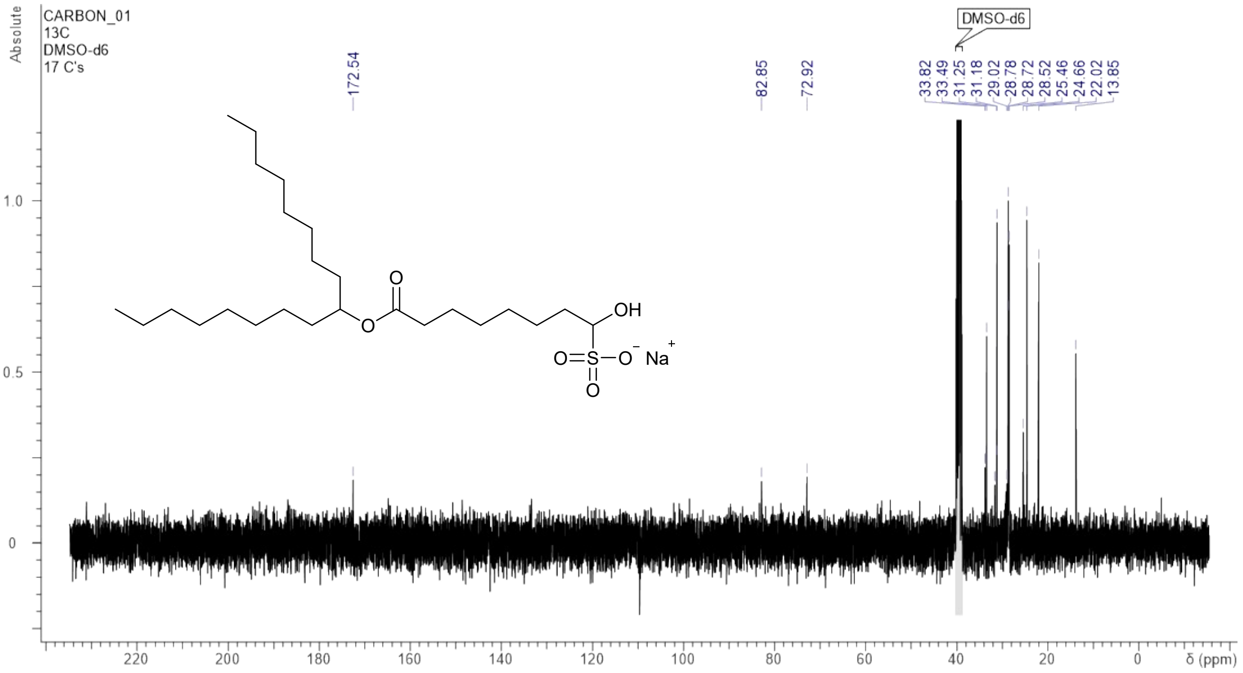

5.1. HPLC-CAD Chromatograms

ALC-0315 **1** From 6-bromohexyl 2-hexyldecanoate **2**:

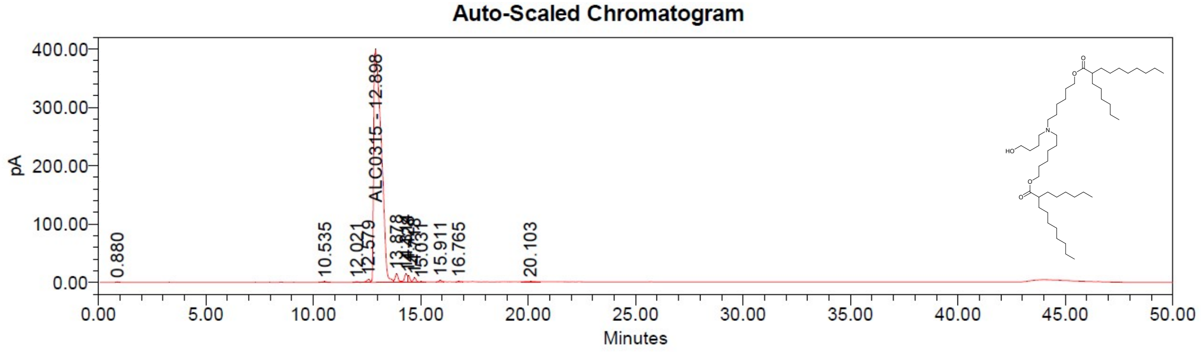

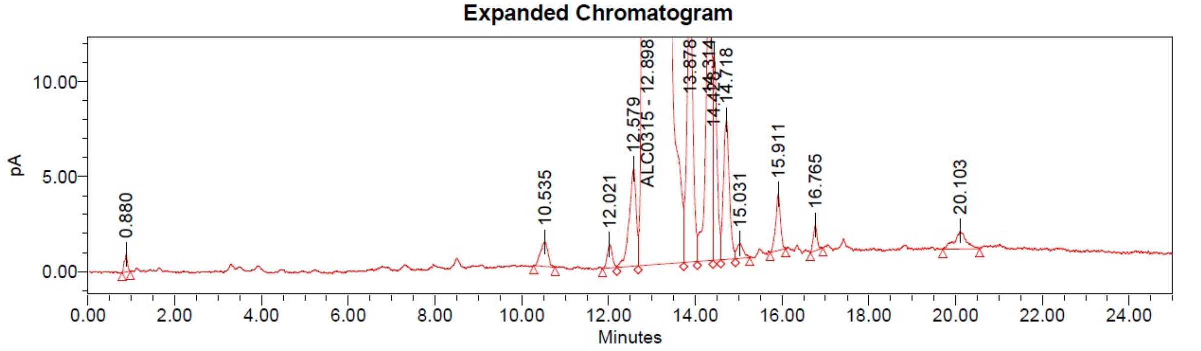

Purity (area %) = 94.9%

ALC-0315 **1** From 6-hydroxyhexyl 2-hexyldecanoate **9**:

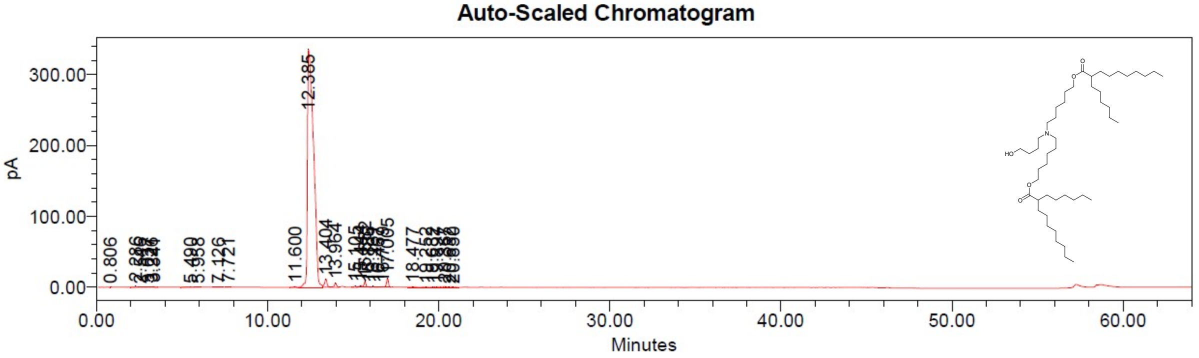

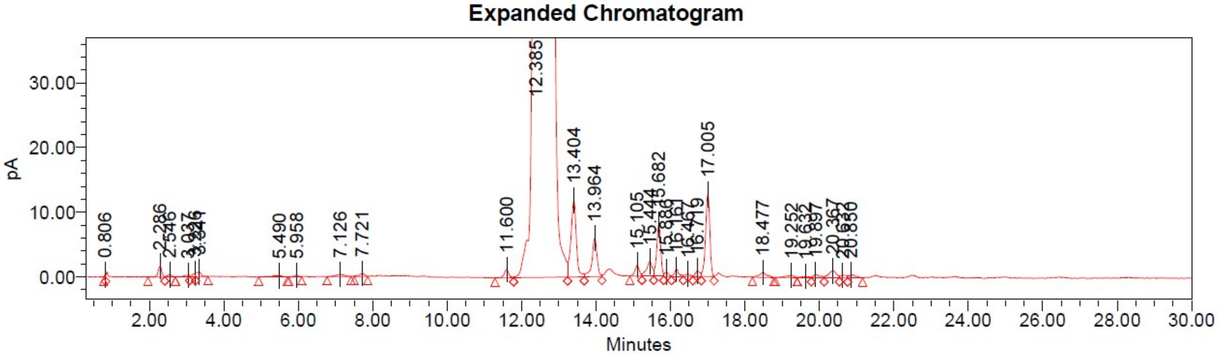

Purity (area %) = 97.3%

SM-102 **6**:

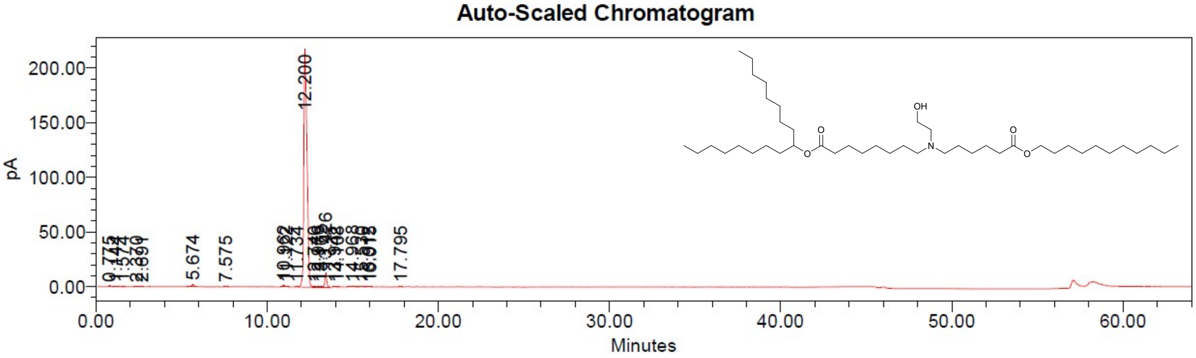

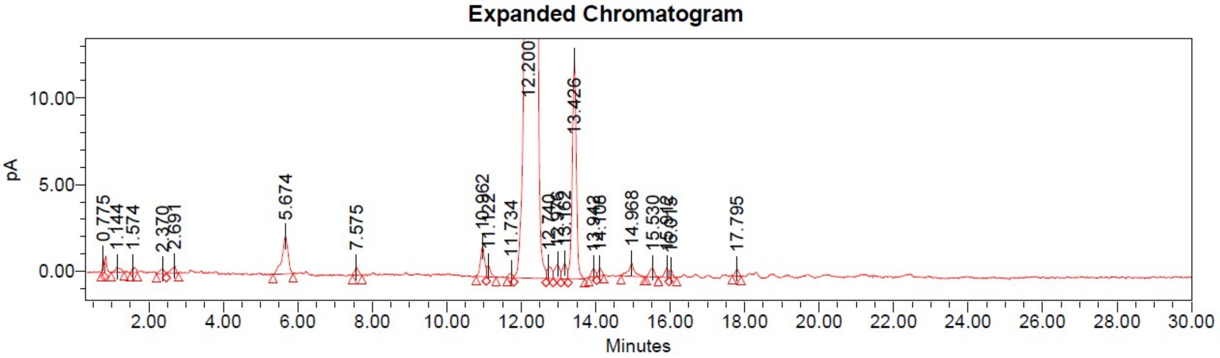

Purity (area %) = 96.0%

References

1 J. B. Wolf, J. Weon Lee, M. B. Plutschack, D. Cambié, A. Seidel-Morgenstern and P. H. Seeberger, *React. Chem. Eng.*, 2024, **9**, 959–966.

2 I. A. Boldyrev, V. P. Shendrikov, A. G. Vostrova and E. L. Vodovozova, *Russ. J. Bioorganic Chem.*, 2023, **49**, 412–415.

3 F. Saadati, S. Cammarone and M. A. Ciufolini, *Chem. – Eur. J.*, 2022, **28**, e202200906.

4 X. Zhang, W. Zhao, G. N. Nguyen, C. Zhang, C. Zeng, J. Yan, S. Du, X. Hou, W. Li, J. Jiang, B. Deng, D. W. McComb, R. Dorkin, A. Shah, L. Barrera, F. Gregoire, M. Singh, D. Chen, D. E. Sabatino and Y. Dong, *Sci. Adv.*, 2020, **6**, eabc2315.

5 M. Sawicki, D. Lecerclé, G. Grillon, B. Le Gall, A.-L. Sérandour, J.-L. Poncy, T. Bailly, R. Burgada, M. Lecouvey, V. Challeix, A. Leydier, S. Pellet-Rostaing, E. Ansoborlo and F. Taran, *Eur. J. Med. Chem.*, 2008, **43**, 2768–2777.

6 WO2023121970A1, 2023.

7 CN114874104A, 2022.

8 Z. Chen, N. Salehi Marzijarani, S. Quirie, G. F. Pirrone, S. M. Dalby, T. Wang, J. Kim, F. Peng and A. J. Fine, *Org. Process Res. Dev.*, 2022, **26**, 525–532.

9 G. Torres, J. A, L. Sii Hong, P. Anchuri, J. M. Stevens, J. E. Tabora, J. Li, A. Borovika, R. P. Adams and A. G. Doyle, EDBO+. Bayesian reaction optimization as a tool for chemical synthesis.

10 A. G. Godfrey and B. Ganem, *Tetrahedron Lett.*, 1990, **31**, 4825–4826.

11 A. B. Smith, K. Basu and T. Bosanac, *J. Am. Chem. Soc.*, 2007, **129**, 14872–14874.

12 A. B. Smith III, T. Bosanac and K. Basu, *J. Am. Chem. Soc.*, 2009, **131**, 2348–2358.

13 N. Kornblum, W. J. Jones and G. J. Anderson, *J. Am. Chem. Soc.*, 1959, **81**, 4113–4114.

14 B. Paisdor and D. Kuck, *J. Org. Chem.*, 1991, **56**, 4753–4759.

15 D. Kuck, *Chem. Ber.*, 1994, **127**, 409–425.

16 D. Kuck, A. Schuster, B. Paisdor and D. Gestmann, *J. Chem. Soc. Perkin 1*, 1995, 721–732.

17 H. Finkelstein, *Berichte Dtsch. Chem. Ges.*, 1910, **43**, 1528–1532.
